# Tissue-resident memory T cells break tolerance to renal autoantigens and orchestrate immune-mediated nephritis

**DOI:** 10.1038/s41423-024-01197-z

**Published:** 2024-07-03

**Authors:** Frederic Arnold, Laurence Kupferschmid, Philipp Weissenborn, Lukas Heldmann, Jonas F. Hummel, Paulina Zareba, Sagar, Manuel Rogg, Christoph Schell, Yakup Tanriver

**Affiliations:** 1 Department of Medicine IV, Medical Center, Faculty of Medicine, University of Freiburg, Freiburg, Germany; 2 Institute of Microbiology and Hygiene, Medical Center, Faculty of Medicine, University of Freiburg, Freiburg, Germany; 3 Institute of Pathology, Medical Center, Faculty of Medicine, University of Freiburg, Freiburg, Germany; 4 Department of Medicine II, Medical Center, Faculty of Medicine, University of Freiburg, Freiburg, Germany

**Keywords:** T cell response, Tissue residency, Renal autoimmunity, Nephritis, CD8, Autoimmunity, Cytotoxic T cells, Lymphocyte differentiation

## Abstract

Immune-mediated nephritis is a leading cause of acute kidney injury and chronic kidney disease. While the role of B cells and antibodies has been extensively investigated in the past, the advent of immune-checkpoint inhibitors has led to a reappraisal of the role of T cells in renal immunology. However, it remains elusive how T cells with specificity for renal autoantigens are activated and participate in immune-mediated nephritis. Here, we followed the fate and function of pathogen-activated autoreactive CD8 T cells that are specific for a renal autoantigen. We demonstrate that recently activated splenic CD8 T cells developed a hybrid phenotype in the context of renal autoantigen cross-presentation, combining hallmarks of activation and T cell dysfunction. While circulating memory T cells rapidly disappeared, tissue-resident memory T cells emerged and persisted within the kidney, orchestrating immune-mediated nephritis. Notably, T cells infiltrating kidneys of patients with interstitial nephritis also expressed key markers of tissue residency. This study unveils how a tissue-specific immune response can dissociate from its systemic counterpart driving a compartmentalized immune response in the kidneys of mice and man. Consequently, targeting tissue-resident memory T cells emerges as a promising strategy to control immune-mediated kidney disease.

## Introduction

Immune-mediated nephritis results from an inappropriate immune response against self-antigens or innocuous substances (e.g., antibiotics). It can cause renal function decline up to end-stage renal disease. To date, a substantial body of knowledge exists regarding antibody-mediated forms of immune-mediated nephritis [1]. In contrast, the role of cellular immunity in the context of immune-mediated kidney disease lacks understanding, even though infiltrates of various immune cells are regularly present in biopsies of affected kidneys. Additionally, the density and composition of inflammatory infiltrates correlate with the deterioration of renal function [2]. Thus, there is a pressing need to decipher cellular immunity in the kidney.

Cytotoxic CD8^+^ T lymphocytes (CTLs) represent one of the most abundant immune cell types within renal infiltrates and there is strong evidence that antigen-specific CTLs play a pivotal role in various entities of immune-mediated nephritis [3]. CTL infiltrates are particularly common in forms of interstitial nephritis (e.g., drug-induced acute interstitial nephritis [AIN] or immune-checkpoint inhibitor [ICI]-related nephritis) and can be found in 15–27% of renal biopsies performed for acute kidney injury [4]. Moreover, various infections or systemic auto-immune diseases can also be associated with interstitial nephritis [5]. Intriguingly, only a minority of patients treated with a particular drug develop AIN, indicative of a continuous and active regulation of local immune responses in the kidney protecting its structural integrity. Similar to the gut, the kidney is constantly exposed to xenobiotic products, which it removes from the circulation. It therefore must balance immune responses to innocuous or self-antigens and pathogens. Hence, CTL-driven immune-mediated disease occurs if central or peripheral tolerance mechanisms fail to protect the host from a self-targeted immune response. The high incidence of immune-mediated nephritis following ICI therapy for malignant disease further underlines the importance of peripheral tolerance mechanisms in protecting the kidneys from autoreactive T cells [6]. Nevertheless, the details of underlying molecular programs defining and controlling this autoreactive state, as well as the complex mechanisms of activation, differentiation, and memory formation of autoreactive CTLs are not well understood. Since nephritis involves a primarily localized immune response, the question arises how local immunity is initiated and maintained in the kidney over time. This also involves the issue of how CTLs that are situated in the interstitium of the kidney gain access to their cognate antigen after its passage through the nephron, from which they are separated by a layer of epithelial cells. Tissue-resident memory T (T_RM_) cells are a potential culprit as they are a local memory population that stably resides in the tissue over time and has been identified in various organs, including the kidneys [7]. T_RM_ cell differentiation is essential in accelerating local pathogen control [8]. However, it is unclear to what extent they also contribute to inducing and maintaining renal autoimmunity [9].

Although, several murine models (e.g., transgenic lupus nephritis, pauci-immune nephritis, immunoglobulin A nephritis, or nephrotoxic nephritis models) have been established to study auto-immune kidney disease, none of these models focuses on the cellular immune response in the kidney [10–13]. Hence, studying details of CTL-driven renal autoimmunity relies on the establishment of novel preclinical models.

In this study, we present a new mouse model for cellular autoimmunity in the kidney, where in vivo activation of adoptively transferred self-reactive CTLs results in auto-immune kidney disease. We utilized this model to dissect the role of self-reactive CTLs, applying single-cell transcriptomics, high-dimensional flow cytometry, and multiplexed immunofluorescence imaging. We could demonstrate that renal autoantigen expression induced a form of split tolerance in pathogen-activated CTLs, if mice were infected with a pathogen that expresses the same autoantigen. This split tolerance was characterized by a rapidly dissipating systemic immune response, while a robust and long-lasting auto-immune response was propagated in the kidney. Within renal infiltrates, we identified a subpopulation of autoreactive T cells that expressed canonical T_RM_ cell markers. These T cells promoted local immunity under the influence of sustained autoantigen cross-presentation. Interestingly, comparable T cell populations, bearing hallmarks of tissue residency, were also found in the kidneys of patients with active nephritis. Hence, our data strongly suggest that these potential T_RM_ cells are crucial for maintaining renal autoimmunity and thus may provide a possible target for novel therapeutic approaches.

## Results

### Targeting glomerular antigen to model cellular autoimmunity in the kidney

**Fig. 1:**
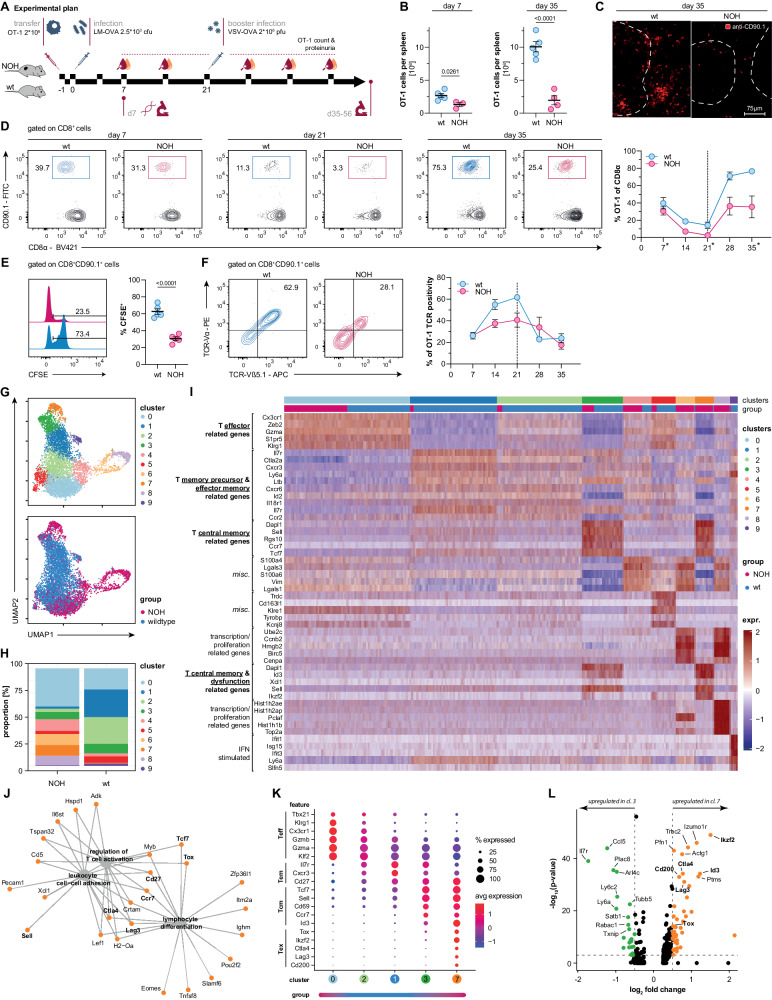
OT-1 cells develop a distinct phenotype in an autoreactive environment. **A** Experimental plan. Initially (d1) 2 × 10^6^ OT-1 cells were i.v. transferred into wt and NOH mice. After 24 h (d0) mice were infected with 2.5 × 10^3^ cfu of OVA-expressing *Listeria monocytogenes* (LM-OVA). After 21 days, mice were reinfected/boostered with 2 × 10^6^ pfu OVA-expressing vesicular stomatitis virus (VSV-OVA). Blood and urine samples were analyzed weekly. Final analysis was performed at early (d7) and a late time points (d35–56). **B** Quantitative flow cytometry analysis depicting less OT-1 cells in the spleens of NOH (red) vs. wt mice (blue) after 7 and 35 days. Total cell numbers were calculated by manually counting lymphocytes of whole organ lysates and multiplying counts with fractions obtained by flow cytometry analysis. **C** Anti-CD90.1 (red channel) immunofluorescence confirming less transferred OT-1 cells in spleens of NOH (right) vs. wt mice (left). **D** Representative flow cytometry plots and summary analysis (*n* = 5 per group) showing quicker decline of transferred OT-1 cells in peripheral blood of NOH mice (red) after primary (d7, d21) and secondary booster infection (d35) compared to wt mice (blue) over time. Dashed line indicating time point of booster infection. **E** CFSE assay demonstrating faster initial proliferation of transferred OT-1 cells in LM-OVA infected NOH (red) compared to wt mice (blue) after 72 h. **F** Representative flow cytometry plots showing expression of T cell receptor (TCR) chains Vα2 and Vβ5.1 prior to reinfection (d21). Time-course of TCR expression (right) demonstrating earlier TCR-internalization in NOH (red) compared to wt mice (blue). Dashed line indicating time point of booster infection. **G** Unsupervised UMAP-clustering of ~7000 OT-1 cell transcriptomes identifying 10 distinct clusters (upper plot). Cells from wt (blue) vs. NOH mice (red) clustered separately (lower plot). **H** Stacked barplots visualizing cluster composition of NOH vs. wt mice. Clusters 0, 4, 6, 7, and 8 mainly defined NOH-derived cells. **I** Annotated heat map of cluster-defining transcripts. Top five transcripts were identified by highest significant avgLog2FC values. **J** GO:BP term enrichment analysis of genes overexpressed in cluster 7. Network depicting linkages of genes and biological processes. **K** Dot plot illustrating the functional classification of clusters 0, 1, 2, 3, and 7 by curated genes associated with T cell effector, memory and inhibitory features. Bar below indicates if clusters were mainly represented by NOH or wt-derived OT-1 cells. **L** Volcano plot depicting differentially expressed genes comparing clusters 3 and 7. Dashed lines indicate log_2_ fold change value of 0.5 and *p* value of 0.05. If not stated differently, bars and whiskers of dot plots representing flow cytometry data depict means and respective standard errors of the mean (SEM). *P* values were calculated using unpaired student’s *t* test. All presented datasets are representative of at least three individual experiments with *n* ≥ 3 mice/group. To obtain single-cell RNA-sequencing data, OT-1 cells were adoptively transferred into wt and NOH mice and isolated 7 days after LM-OVA infection as depicted in (**A**). Splenocytes of four mice (two wt and two NOH each) were harvested and FACS-sorted to isolate OT-1 cells. Cells were labeled with antibody bound hashtag oligos and pooled together for sequencing (detailed information provided in “Materials and Methods” section)

To unravel cytotoxic CD8^+^ T lymphocyte (CTL)-driven autoimmunity in the kidney, we established a new murine model, based on NOH (nephrin, chicken ovalbumin [OVA], hen egg lysozyme [HEL]) mice, expressing membrane bound OVA on the surface of podocytes (Supplementary Fig. S1A, B) [14]. Next, congenically (CD90.1) marked high affinity OVA_257–264_/H-2K^b^-specific T cell receptor transgenic CD8^+^ T cells (OT-1 cells) were adoptively transferred into C57BL/6-NOH (NOH) and C57BL/6-wildtype (wt) mice (d1) [15, 16]. OT-1 cells transferred into NOH hosts quickly perished after 1 week (Supplementary Fig. S1C). Thus, subclinical infection with 2.5 × 10^3^ cfu OVA-expressing *Listeria monocytogenes* (LM-OVA) was performed 24 h after cell transfer (d0), promoting in vivo activation of transferred OT-1 cells [17]. To sustain cellular immunity over time, mice received a subclinical booster infection with 2 × 10^6^ pfu of OVA-expressing vesicular stomatitis virus (VSV-OVA) on day 21 [18, 19]. Weekly blood and urine analyses were performed on NOH and wt mice to monitor transferred OT-1 cells and proteinuria in the cohorts. For endpoint analyses, mice were sacrificed at early (d7; after primary infection) and late time points (d35–56; after booster infection). A detailed experimental plan is illustrated in Fig. 1A.

### Autoantigen expression facilitates differentiation of a distinct CTL phenotype

Transferred OT-1 cells were detectable in all mice after transfer and infection, indicating a robust cellular immunization model. Interestingly, when compared to wt mice, the total number of OT-1 cells detected in secondary lymphoid organs of NOH mice was significantly lower after 7 (1.3 [±0.3] × 10^6^ vs. 2.7 [±0.3] × 10^6^ OT-1 splenocytes; *P* = 0.0261) and 35 days (2.0 [±0.7] × 10^6^ vs. 10.1 [±0.8] × 10^6^ OT-1 splenocytes, *P* < 0.001) (Fig. 1B, C and Supplementary Fig. S2A, B). Besides lower OT-1 frequencies, a more rapid decline of transferred cells after primary infection (day 21: 2.5 [±1.3]% vs. 14.2 [±3.7]%, *P* = 0.0114), as well as a limited re-expansion after booster infection (day 35: 35.5 [±3.0]% vs. 76.5 [±12.6]%; *P* = 0.0132) was detected in peripheral blood of NOH vs. wt mice (Fig. 1D). These findings demonstrate a curtailed systemic immune response in NOH mice as a result of autoantigen expression in the kidney. To better understand how renal autoantigen expression affects early activation and division kinetics, we adoptively transferred CFSE-labeled OT-1 cells into wt and NOH mice and infected them as described. Intriguingly, OT-1 cells in NOH mice divided much faster within the first 72 h after transfer and infection than OT-1 cells in wt mice (30.4 [±2.1]% vs. 62.6 [±3.3]% of CFSE^+^ OT-1 cells, *P* < 0.0001; Fig. 1E). Hence, we conclude that renal autoantigen is recognized by adoptively transferred OT-1 cells and fuels their early activation during infection with pathogens expressing the same antigen. Continuous autoantigen recognition in NOH hosts is further corroborated by sustained T cell receptor internalization of OT-1 cells from NOH mice (Fig. 1F) [20].

To characterize the systemic immune response and differentiation of autoantigen-specific CTLs in the context of concurrent renal antigen cross-presentation, we performed single-cell RNA sequencing of OT-1 cells, isolated from splenocyte suspensions of NOH and wt mice 7 days after initial transfer and infection. Unsupervised Uniform Manifold Approximation and Projection (UMAP) clustering of ~7000 OT-1 cells resulted in 10 clusters. Interestingly, OT-1 cells isolated from NOH (red) and wt mice (blue) clustered separately, indicating a distinct transcriptional profile of CTLs in the context of an auto-immune environment (Fig. 1G). Over 80% of NOH-derived OT-1 cells accumulated in clusters 0, 4, 6, 7, and 8, while cells from wt mice were mainly found in clusters 0, 1, 2, and 3 (Fig. 1H). Clusters were classified by their five most upregulated transcripts. We could identify subpopulations of OT-1 cells from NOH mice, expressing proliferation-associated genes, indicating higher proliferative activity of naïve autoreactive OT-1 cells in the NOH environment (clusters 6 and 8; Fig. 1I), which was well in line with CFSE division profiles (Fig. 1E). Properties of terminal T effector differentiation (upregulation of e.g., *Klrg1*, *Cx3cr1*, *Zeb2*, *S1pr5*, and *Gzma*) could be found in OT-1 cells derived from both NOH and wt mice, with a higher relative proportion in NOH mice (cluster 0; Fig. 1H, I) [21–23]. By contrast, upregulation of T memory (precursor) associated transcripts (e.g., *Il7r*, *Cxcr3*, *Id2*) was mainly found in OT-1 cells isolated from wt mice (clusters 1 and 2, Fig. 1I) [24]. This may indicate a significantly altered T effector and T memory cell differentiation in NOH mice due to permanent antigen exposure. Pseudotemporal ordering of the sequencing data revealed a branched trajectory, suggesting differentiation of T effector and T memory cells originating from common stem cell-like precursors. NOH-derived OT-1 cells mainly clustered at the tips of the trajectories, corroborating a further advanced, distinct differentiation of both T effector and T memory populations in the context of persistent autoantigen recognition (Supplementary Fig. S3A). Interestingly, two clusters shared properties of T central memory (T_CM_) differentiation (e.g., upregulation of *Sell*, *Ccr7*, *Tcf7*) [25, 26]. One mainly contained wt-derived OT-1 cells, the other one cells from NOH mice (clusters 3 and 7, respectively, Fig. 1I). Functional enrichment analysis on differentially expressed genes (DEGs) of the NOH-derived cluster 7 divulged an upregulation of genes associated with lymphocyte differentiation and regulation of T cell activation (Fig. 1J). Further analysis of feature expression regarding curated effector, memory and inhibitory signatures of clusters 0, 1, 2, 3, and 7 (Fig. 1K) and comparing DEGs of clusters 7 and 3 (Fig. 1L) revealed a distinct T memory phenotype of NOH-derived OT-1 cells in cluster 7: while cells of cluster 3 predominantly expressed T memory associated transcripts (e.g., *Sell*, *Il7r*, *Tcf7*, *Ccr7*), NOH-derived OT-1 cells of cluster 7 also expressed markers associated with T cell dysfunction (e.g., *Tox*, *Ikzf2*, *Ctla4*, *Lag3*, *Cd200*) [24, 27]. Interestingly, *Id3*, a T memory marker delineating precursors of exhausted T cells was also more highly expressed in cluster 7 (Fig. 1K, L and Supplementary Fig. S3B–D) [28]. A unique phenotype of OT-1 memory cells in NOH mice was further delineated by complementary gene set enrichment (GSEA) and feature expression analyses (Supplementary Fig. S4A, B). Hence, the presented analysis supports a model in which splenic OT-1 cells from NOH mice display a hybrid phenotype, comprising both features of T memory formation and T cell dysfunction.

**Fig. 2:**
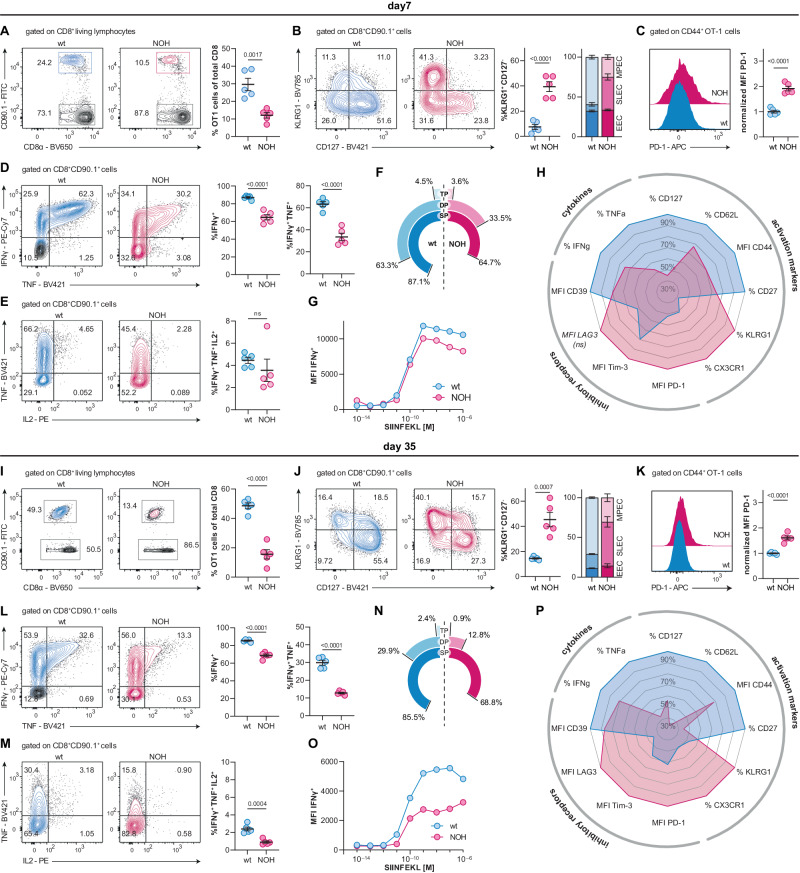
Autoreactive OT-1 cells express more inhibitory markers and produce less cytokines, 7 (**A**–**H**) and 35 days (**I**–**P**) after adoptive cell transfer. Representative flow cytometry plots discriminating transferred OT-1 cells from native CD8^+^ cells (CD90.1^−^) in splenocyte suspensions. Dot plot (right) shows significantly lower OT-1 cell fractions in NOH (red) compared to wt mice (blue) on day 7 (**A**) and day 35 (**I**). Representative flow cytometry plots illustrating KLRG1 and CD127 expression of transferred OT-1 cells. Dot plot and stacked bar plot depicting higher percentages of KLRG1^+^ CD127^−^ short-lived effector OT-1 cells, compared to KLRG1^−^ CD127^+^ memory precursor OT-1 cells in NOH (red) vs. wt mice (blue) on day 7 (**B**) and day 35 (**J**). Representative histograms illustrating PD-1 expression of transferred OT-1 cells. Dot plot comparing normalized mean fluorescent intensities (MFIs) of the PD-1 signal, showing higher PD-1 expression in OT-1 cells from NOH (red) vs. wt mice (blue) on day 7 (**C**) and day 35 (**K**). Flow cytometry plots illustrating interferon-γ (IFNγ) and tumor necrosis factor (TNF) production of transferred OT-1 cells after in vitro restimulation with 10^−6^ M SIINFEKL peptide; cytokine excretion blocked with brefeldin A. Dot plots showing lower fractions of IFNγ^+^ and IFNγ^+^TNF^+^ double-positive OT-1 cells in NOH (red) vs. wt mice (blue) on day 7 (**D**) and day 37 (**L**). Flow cytometry plots illustrating tumor necrosis factor (TNF) and IL2 production of transferred IFNγ^+^-OT-1 cells after in vitro restimulation. Dot plots indicating lower fractions of IL2^+^IFNγ^+^TNF^+^ triple positive OT-1 cells in NOH (red) vs. wt mice (blue) on day 7 (**E**) and day 35 (**M**). Radial charts comparing cytokine production of OT-1 cells in NOH (red) vs. wt mice (blue). SP single positive, (IFNγ^+^); DP double positive, (IFNγ^+^TNF^+^); TP triple positive, (IL2^+^IFNγ^+^TNF^+^) on day 7 (**F**) and day 35 (**N**). Functional avidity testing demonstrated that higher SIINFEKL concentrations could not increase the capability of IFNγ production in OT-1 cells from NOH mice (red) on day 7 (**G**) and day 35 (**O**). Spider graphs summarizing relative expression of activation markers, inhibitory receptors and cytokine production on day 7 (**H**) and day 35 (**P**). Bars and whiskers of dot plots depict means and respective standard errors of the mean (SEM). *P* values were calculated using unpaired student’s *t* test. All presented datasets are representative of at least three individual experiments with *n* = 5 mice per group. Normalized Median Fluorescence Intensities (MFIs) were calculated by dividing the MFIs of each sample by the mean MFI of the respective wt group

Next, multi-color flow cytometry analysis was performed to explore the differentiation phenotype and evaluate the cytotoxic potential of autoreactive OT-1 cells. As indicated by histological analysis (Fig. 1B, C), lower percentages of transferred OT-1 cells (12.1 vs. 29.6%, *P* = 0.0017) could be retrieved from NOH mice at day 7 (Fig. 2A). OT-1 cells from NOH mice displayed a strong skewing toward a T effector phenotype with elevated frequencies of KLRG-1^+^ (39.4 vs. 7.4%, *P* < 0.0001), CD62L^−^CD44^+^ (48.7 vs. 34.1%, *P* = 0.0027), and CX3CR1^+^ (41.9 vs. 21.5%, *P* = 0.0005; Fig. 2B and Supplementary Fig. S5A, B). Besides pronounced effector characteristics, OT-1 cells from NOH mice expressed higher levels of the inhibitory receptors programmed death-1 (PD-1; normalized MFI 1.0 vs. 1.9, *P* < 0.0001) and T cell immunoglobulin mucin-3 (Tim-3; normalized MFI 1.0 vs. 1.3, *P* = 0.0395), even at an early time point (Fig. 2C and Supplementary Fig. S5C–F). To assess the cytotoxic capacity of OT-1 cells, splenocytes were restimulated in vitro with their cognate antigen (SIINFEKL): after restimulation, fewer OT-1 cells from NOH vs. wt mice were capable of producing the pro-inflammatory cytokines IFN-γ (64.7 vs. 87.1%, *P* < 0.0001), TNF (33.5 vs. 63.3% double positive, *P* < 0.0001), and IL2 (non-significant trend at d7; Fig. 2D–F). Exposing OT-1 cells to increasing amounts of SIINFEKL and measuring cytokine production in vitro, showed lower functional avidity in OT-1 cells from NOH mice, which could not be compensated for with higher antigen concentrations (data shown for IFN-γ; Fig. 2G). Figure 2H summarizes the immune phenotype of OT-1 cells isolated from both NOH and wt mice on day 7. The results align with our single-cell data, supporting the conclusion of an aberrant differentiation in the context of autoantigen recognition.

Autoreactive OT-1 cells bear the potential of re-expansion upon systemic reencounter with the autoantigen. As demonstrated histologically and in the time-course analysis of peripheral blood (Fig. 1B–D), OT-1 proliferation was curtailed in NOH mice after booster infection with VSV-OVA (15.6 vs. 48.6%, *P* < 0.0001, Fig. 2I). The above described hybrid phenotype of NOH-derived OT-1 cells was even more striking after re-challenge with VSV-OVA. Skewing toward effector differentiation of NOH-derived OT-1 cells was even more pronounced at day 35 (KLRG1^+^ 45.3 vs. 14.5%, *P* = 0.0007; CD62L^−^ 88.5 vs. 66.7%, *P* < 0.0001; CX3CR1^+^ 71.7 vs. 35.2%, *P* < 0.0001; Fig. 2J and Supplementary Fig. S5G, H). Additionally, more inhibitory receptors were upregulated in OT-1 cells of NOH vs. wt mice at the late time point (Fig. 2K and Supplementary Fig. S5I–L). While the capability of IFN-γ production was maintained in NOH-derived OT-1 cells at day 35 (IFN-γ^+^: 68.8 vs. 85.5%, *P* < 0.0001), fractions of IFN-γ^+^TNF^+^-double positive (12.8 vs. 29.9%, *P* < 0.0001) and INF-γ^+^TNF^+^IL2^+^-triple positive (0.9 vs. 2.4%, *P* = 0.0004) cells were generally lower at the late time point (Fig. 2L–N), and the reduced functional avidity in NOH vs. wt OT-1 cells was still apparent (Fig. 2O). Figure 2P summarizes the immune phenotype of OT-1 cells at day 35. Comparable results could be observed when a higher LM-OVA dose (250,000 cfu) was administered as a booster infection (homologous prime-boost system) instead of VSV-OVA (Supplementary Fig. S6).

In summary, autoantigen exposure resulted in a restricted systemic immune response in NOH mice: First, a more rapid decline of peripheral OT-1 cells was induced. Second, it imposed the development of a stable hybrid OT-1 phenotype in NOH mice, which was characterized by both T memory features and T inhibitory traits. Lastly, while higher proportions of effector OT-1 cells differentiated in NOH mice, their capacity to produce cytokines was notably restricted.

### Autoreactive CTLs can induce a renal phenotype

**Fig. 3:**
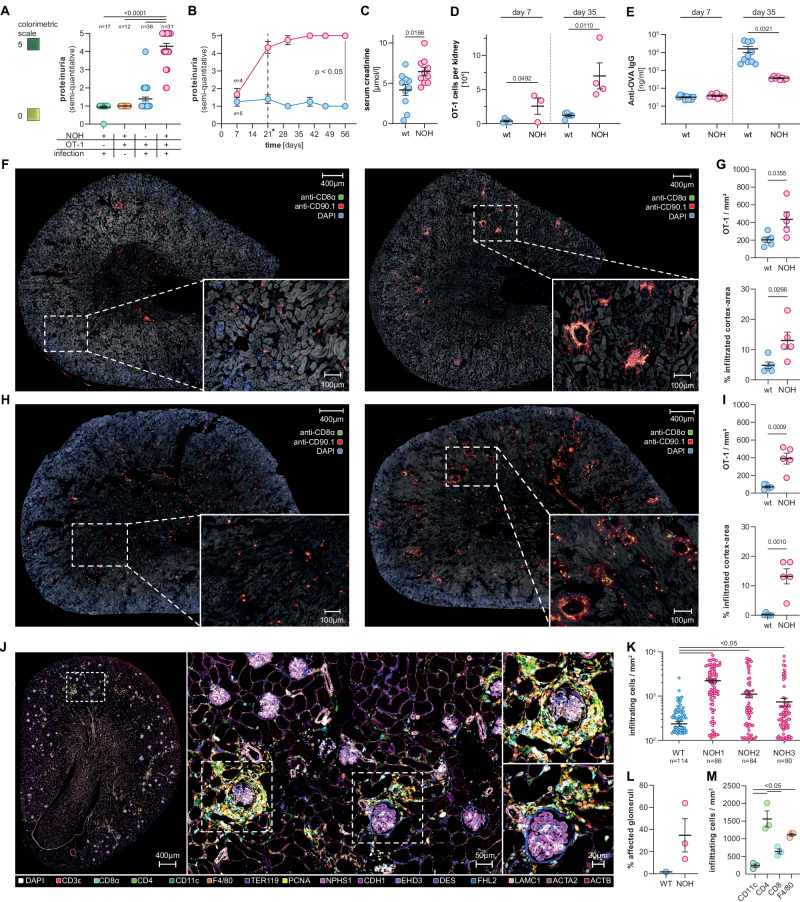
Autoreactive OT-1 cells induce a renal phenotype. **A** Dot plot illustrating semi-quantitative proteinuria assessed via colorimetric dipstick assays after 35 days. NOH mice (red) develop significant proteinuria after OT-1 cell transfer and infection, compared to wt mice (blue) and NOH mice after either infection (green) or OT-1 transfer (yellow). **B** Time-course showing persistent proteinuria in NOH mice (red). Dashed line indicates time point of booster infection. **C** Dot plot showing higher serum creatinine levels in NOH (red) vs. wt (blue) mice after 35 days. **D** Quantitative flow cytometry analysis depicting more OT-1 cells in the kidney of NOH (red) vs. wt mice (blue) after 7 (left) and 35 (right) days. Total cell numbers were calculated by manually counting lymphocytes of whole organ lysates and multiplying counts with fractions obtained by flow cytometry analysis. **E** Dot plot comparing anti-OVA IgG levels in serum of NOH (red) vs. wt (blue) mice. Lower IgG levels were detected in NOH vs. wt mice after 35 days, indicating a curtailed humoral immune response. **F** Immunofluorescence of whole kidney sections, labeled with FITC-anti-CD8α- (green channel) and PE-anti-CD90.1-antibodies (red channel), depicting the OT-1 cell infiltration in wt (left) and NOH mice (right) after 7 days. **G** Dot plots showing higher OT-1 cell density/mm^2^ (upper graph) and percentage of infiltrated cortex area (lower graph) in NOH (red) vs. wt mice (blue) after 7 days. **H** Immunofluorescence of whole kidney sections, labeled with FITC-anti-CD8α- (green channel) and PE-anti-CD90.1-antibodies (red channel), depicting the OT-1 cell infiltration in wt (left) and NOH mice (right) after 35 days. **I** Dot plots showing higher OT-1 cell density/mm^2^ (upper graph) and percentage of infiltrated cortex area (lower graph) in NOH (red) vs. wt mice (blue) after 35 days. **J** 16-plex 4i immunofluorescence of whole kidney sections, labeling immune cell entities and structural markers after 35 days. **K** Dot plot showing a higher number of immune cells per infiltrated periglomerular area in NOH (red) mice. *n* depicts the number of analyzed glomeruli/sample. Comparison of three NOH mice (red) to wt control (blue). **L** Dot plot illustrating fractions of affected glomeruli/whole kidney section. **M** Dot plot illustrating immune-cell composition of periglomerular infiltrates. Bars and whiskers of dot plots depict means and respective standard errors of the mean (SEM). *P* values were calculated using unpaired student’s *t* test or one-way ANOVA with post-hoc Tukey’s multiple comparisons method if two or more groups were analyzed. All presented datasets are representative of at least three individual experiments with *n* ≥ 3 mice/group

Despite the abrogated activation and differentiation of OT-1 cells, NOH mice developed significant proteinuria after OT-1 transfer and infection compared to wt mice, indicating renal pathology driven by podocyte-specific OVA expression. Neither adoptive OT-1 transfer nor prime-boost infection with OVA-expressing pathogens alone could induce a detectable renal phenotype (Fig. 3A). Pathological proteinuria in NOH mice was measurable beginning 14–21 days after OT-1 transfer and remained elevated over the entire observational period (Fig. 3B). Accordingly, serum creatinine levels were higher in NOH vs. wt mice after 35 days (6.5 [±0.5] vs. 4.2 [±0.7] µmol/l; *P* = 0.0166; Fig. 3C). Both findings indicate renal damage in the context of CTL-driven autoimmunity.

In contrast to limited OT-1 expansion in the periphery, transferred cells accumulated in the kidneys of NOH mice, where the autoantigen is expressed. Significantly more OT-1 cells were detected in kidneys of NOH vs. wt mice, 7 (2.6 [±1.2] × 10^4^ vs. 0.4 [±0.1] × 10^4^; *P* = 0.0492) and 35 days (7.0 [±1.9] × 10^4^ vs. 1.2 [±0.2] × 10^4^; *P* = 0.0110) after transfer (Fig. 3D). Although OVA-specific immunoglobulin G levels remained lower in NOH vs. wt mice (Fig. 3E), humoral immune response may additionally contribute to the observed phenotype.

We performed histopathological analysis of whole kidney sections to further characterize renal infiltrations. Tissues were stained for CD8 and the congenic marker CD90.1 to label transferred OT-1 cells in host kidneys of NOH and wt mice. Seven days (early time point) after OT-1 transfer and primary LM-OVA infection, only a few OT-1 cells were randomly scattered throughout kidneys of wt mice, whereas dense OT-1 infiltrates with predominantly periglomerular and proximal peritubular localization could be detected in kidneys of NOH mice (Fig. 3F). Semi-automated cell type identification and infiltrate detection (Supplementary Fig. S7 and “Materials and Methods” section) confirmed higher numbers of infiltrating OT-1 cells in kidney sections of NOH vs. wt mice (435 [±85] vs. 204 [±33] OT-1 cells/mm^2^; *p*= 0.0355) and larger infiltrated cortex areas (13.0 [±2.8] vs. 4.8 [±1.1]% of infiltrated cortex area; *p* = 0.0266) (Fig. 3G). Corresponding to chronic proteinuria, histopathological analysis on day 35 revealed persistent infiltration (390 [±61] vs. 70 [±12] OT-1 cells/mm^2^; *P* = 0.0009 and 13.2 [±2.6] vs. 0.2 [±0.2]% of infiltrated cortex area; *P* = 0.0010) in kidneys of NOH mice. As opposed to peripheral OT-1 counts, the number of renal OT-1 cells declined in wt mice over time (Fig. 3H, I). Complementary brightfield histology of both time points corroborated these findings (Supplementary Fig. S8A, B). Additional in-depth histopathological analysis on day 35 also revealed features of structural kidney damage in NOH mice (Supplementary Fig. S8C–E).

As expected, neither an isolated (prime-boost) infection nor an isolated adoptive OT-1 transfer was sufficient to induce renal CTL infiltrations (Supplementary Fig. S9A, B). By contrast, comparably dense OT-1 infiltrations were also observed in the context of a homologous prime-boost system, applying a higher LM-OVA dose (250,000 cfu) instead of VSV-OVA as booster infection on day 21 (Supplementary Fig. S10A, B).

To characterize the structure and composition of renal infiltrates, multiplex immunofluorescence staining was performed, targeting a total of 16 different immune cell and structural tissue markers (Fig. 3I and Supplementary Fig. S11). Periglomerular infiltrates appeared significantly denser (1354 [±118] vs. 237 [±32] immune cells/mm^2^, *P* < 0.05; Fig. 3K) and more glomeruli were affected in NOH vs. wt mice (Fig. 3L). As expected, infiltrates were composed by several different immune cell entities. Besides CD8^+^ CTLs (641 [±72] cells/mm^2^), CD4^+^ T cells (1561 [±227] cells/mm^2^), CD11c^+^ dendritic cells or tissue macrophages (235 [±47] cells/mm^2^), and F4/80^+^ macrophages (1111 [±41] cells/mm^2^) could be detected within the periglomerular infiltrates (Fig. 3M).

Collectively, activated autoreactive OT-1 cells could initiate renal autoimmunity in NOH mice, despite the limited systemic cellular immune response in presence of autoantigen expression.

### Glomerullary shedded antigens are presented in the periglomerular space

Since OVA-expressing podocytes in NOH mice reside within the enclosed Bowman’s capsule, they are not directly accessible by immune cells [29]. Hence, we wondered how OT-1 cells are able to sense their cognate antigen in NOH mice and why infiltrates largely surrounded glomeruli and proximal tubules.

**Fig. 4:**
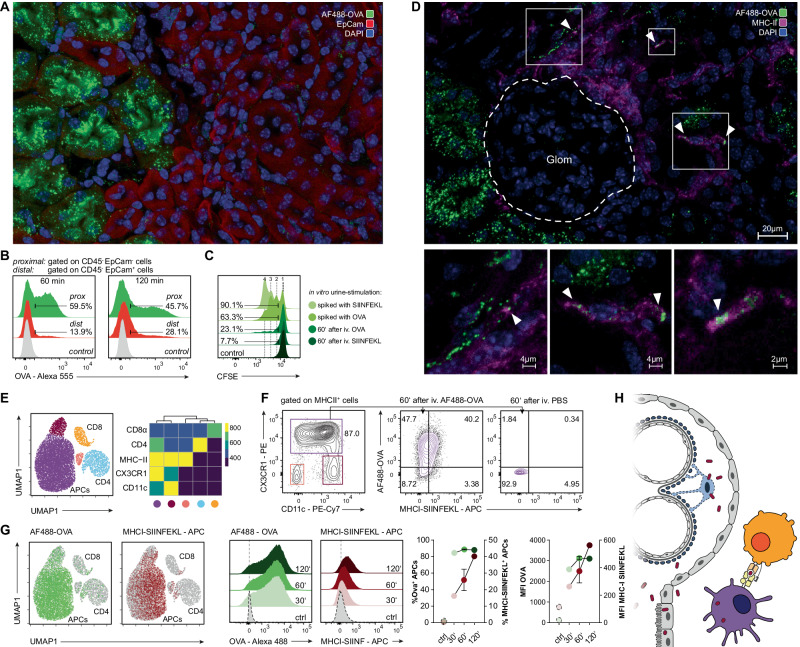
Uptake and presentation of ovalbumin in the kidney. **A** Wt kidney section 60 min after AF488-OVA injection and PBS-perfusion through the renal artery. Representative immunofluorescence showing OVA (green channel) accumulation in proximal tubular cells, identified by low signal of co-staining with anti-CD326 (EpCam)-antibody (red channel). **B** FACS analysis of proximal (CD45^−^EpCam^low^) vs. distal (CD45^−^EpCam^hi^) renal tubular epithelial cells, 60 (left) and 120 min (right) after AF555-OVA injection, demonstrating the OVA uptake in proximal tubular segments. Control: CD45^−^EpCam^low^ renal cells 60 min after PBS injection. **C** In vitro stimulation of CFSE-labeled OT-1 cells for 72 h, demonstrating high effectiveness of OVA uptake in proximal tubular cells. Division rates of OT-1 T cells stimulated with urine of wt mice taken 60 min after i.v. OVA- or SIINFEKL injection were compared to positive controls of OT-1 CD8^+^ T cells stimulated with urine spiked with OVA protein or SIINFEKL peptide. **D** Wt kidney section 60 min after AF488-OVA injection (green channel) and PBS-perfusion through the renal artery; co-staining against MHCII. Representative immunofluorescence demonstrating OVA uptake (green channel; ►) in periglomerular MHCII^+^-antigen-presenting cells (magenta channel). **E** Unsupervised clustering of kidney cells (dimensionality reduction with UMAP and cluster detection with FlowSOM algorithm applied on flow cytometry data), characterizing the renal immune cell compartment (left) and heat map showing relative marker expression of detected clusters (right). **F** Representative flow cytometry plots of kidney cell suspensions 60 min after AF488-OVA injection, demonstrating OVA uptake and SIINFEKL-cross-presentation via MHCII^+^CX3CR1^hi^CD11c^int^ antigen-presenting cells (central plot). Control: 60 min after PBS injection (right plot). **G** Superimposition of AF488-OVA (green, left dot plot) and MHCI-SIINFEKL signals (red, right dot plot) on UMAP, demonstrating processing and cross-presentation of injected OVA in antigen-presenting cells (APCs). Histograms illustrating AF488-OVA (green, left histogram plot) and MHCI-SIINFEKL (red, right histogram plot) signals in APCs, 30, 60, and 120 min after AF488-OVA injection. Graphs on the right comparing percentages (left graph) and MFIs (right graph) of OVA^+^ (green) and MHCI-SIINFEKL^+^ (red) over time. **H** Schematic, illustrating cross-presentation of podocyte antigens: dying podocytes release antigens/epitopes (e.g., OVA) within Bowman’s capsule. They are transcellularly transported in the periglomerular space by proximal tubular epithelial cells. Endocytosis and antigen presentation by APCs can initiate adaptive immunity within the kidney

To assess cross-presentation of antigens that pass through the nephron, fluorescent dye tagged OVA was injected intravenously (i.v.) into wt mice. With a molecular size of ~45 kDa, the conjugate is filtered through the glomerular slit diaphragm. Immunofluorescence imaging of kidney sections 60 min after conjugate injection depicted bright OVA signals in brush borders and apical poles of proximal tubular epithelial cells (PTECs; Fig. 4A). To identify PTECs, anti-EpCam-staining was performed, mainly labeling distal tubular and collecting duct epithelial cells [30]. Quantification of OVA signals by multi-color flow cytometry confirmed initial OVA uptake in proximal, rather than distal tubular or collecting duct epithelial cells (Fig. 4B). Signal intensity in proximal tubular cells slowly declined over time, most likely due to further procession and degradation of the protein. Tubular OVA uptake appeared very effective, with almost all filtrated proteins being taken up by PTECs. To test the efficacy of protein uptake, in vitro antigen stimulation of OT-1 cells with urine of mice 60 min after OVA or SIINFEKL injection was performed. Urine from these mice was not sufficient to induce relevant OT-1 proliferation, compared to urine from control mice that was spiked with OVA or SIINFEKL (Fig. 4C).

Co-staining the tissue sections with anti-MHCII-antibody revealed OVA uptake in MHCII^+^ antigen-presenting cells (APCs) in the interstitium, indicating transcytosis through PTECs (Fig. 4D). To confirm uptake and cross-presentation via APCs, renal immune cells were classified by unsupervised clustering and cluster identification of flow cytometry data from kidney single-cell suspensions (Fig. 4E). MHCII^+^CX3CR1^hi^CD11c^int^ APCs were identified to accumulate fluorescence dye tagged OVA. Moreover, these APCs also cross-presented OVA in the periglomerular and proximal peritubular space. This was demonstrated by flow cytometry staining with the monoclonal antibody 25-D1.16 that recognizes OVA-derived SIINFEKL bound to the MHC class I molecule H2-K^b^ (Fig. 4F). We found that OVA and MHCI-SIINFEKL signals were both enriched in the APC cluster (Fig. 4G). Temporal analysis showed quick OVA uptake in APCs, with signals plateauing 30–60 min after injection. Accordingly, antigen uptake, processing, and cross-presentation were time delayed with signals constantly increasing during the observational period of 120 min (Fig. 4G).

Consequently, our results promoted a model for intrarenal antigen cross-presentation: antigens that are either filtered or shedded within the glomerulus are most likely taken up by PTECs and from there released into the renal interstitium. Here, surveilling APCs can capture, process, and render the antigens accessible to CTLs. Figure 4H illustrates the concepts of this model.

### Autoantigen promotes formation of potential T_RM_ cells fueling local autoimmunity

**Fig. 5:**
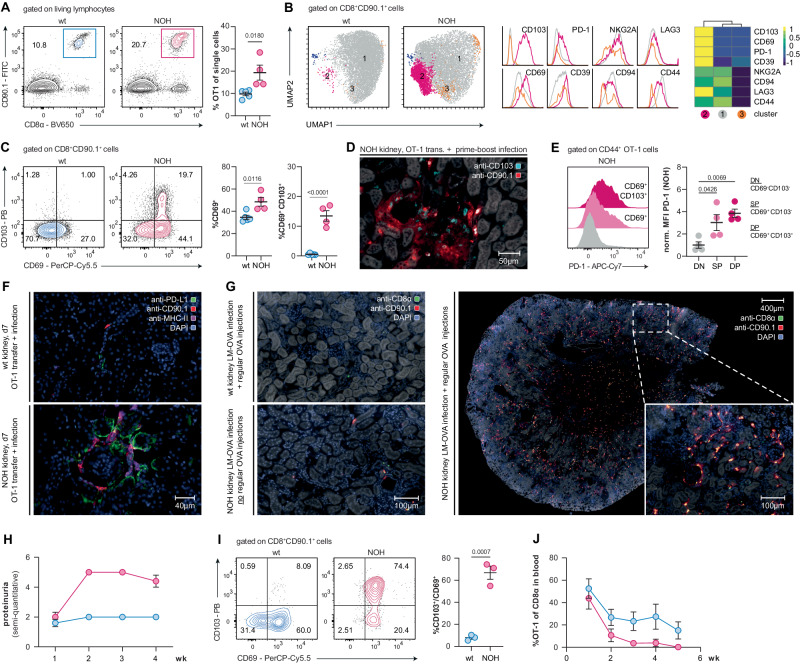
Autoreactive OT-1 cells accumulate in NOH-kidneys originating a potential T_RM_ subpopulation. **A** Representative flow cytometry plots identifying transferred OT-1 cells in kidney single-cell suspensions. Dot plot (right) showing a significantly higher percentage of OT-1 cells in kidneys of NOH (red) compared to wt mice (blue) on day 35. **B** Unsupervised clustering of kidney cells (dimensionality reduction with UMAP and cluster detection with FlowSOM algorithm applied on flow cytometry data) depicting a T_RM_ OT-1 cell subpopulation in kidneys of NOH mice (purple cluster [2]; >100 cells required per defined cluster). Marker-specific histograms and summarizing heatmap showing expression of T_RM_ markers CD103 and CD69, as well as higher levels of inhibitory makers (PD-1, CD39, NKG2A, CD94, LAG3) in cells of the purple cluster (2). **C** Representative flow cytometry plots illustrating CD69 and CD103 expression of renal OT-1 cells on day 35 after heterologous prime-boost infection. Dot plots depicting a higher percentage of CD69^+^ cells (left) and double positive CD69^+^CD103^+^ OT-1 T_RM_ cells (right) in kidneys of NOH (red) vs. wt mice (blue). **D** Immunofluorescence of kidney section, labeled with APC-anti-CD103- (blue channel) and PE-anti-CD90.1-antibodies (red channel), depicting OT-1 T_RM_ cells in periglomerular infiltrates of NOH mice. **E** Representative histograms illustrating PD-1 expression of double positive (CD69^+^CD103^+^), single positive (CD69^+^CD103^−^) and double negative (CD69^−^CD103^−^) renal OT-1 cells in NOH mice on day 35. Dot plot comparing normalized mean fluorescent intensities (MFIs) of the PD-1 signal, showing higher PD-1 expression in double positive (CD69^+^CD103^+^) vs^.^ single positive (CD69^+^CD103^−^) and double negative (CD69^−^CD103^−^) renal OT-1 cells. **F** Representative immunofluorescence of wt (upper picture) and NOH (lower picture) kidney sections, showing elevated PD-L1 expression (green channel) in proximal tubular epithelial cells of infiltrated areas. Co-staining for transferred OT-1 cells (red channel) and renal APCs (magenta channel). **G** Immunofluorescence of kidney sections, labeled with FITC-anti-CD8α- (green channel) and PE-anti-CD90.1-antibodies (red channel), demonstrating that renal OT-1 infiltration can be detected in NOH mice on day 35 after primary infection, followed by weekly OVA injections (right). No infiltrates were detected in wt mice after infection and weekly OVA injections (upper left) and NOH mice after primary infection alone (lower left). **H** Time-course showing persistent proteinuria in NOH mice (red) after primary infection, followed by weekly OVA injections. **I** Representative flow cytometry plots illustrating CD69 and CD103 expression of renal OT-1 cells on day 35 after primary infection and weekly OVA injections. Dot plots depicting a higher percentage of double positive CD69^+^CD103^+^ OT-1 T_RM_ cells (right) in kidneys of NOH (red) vs. wt mice (blue). **J** Summary analysis (*n* = 3/group) showing a decline of transferred OT-1 cells in peripheral blood of NOH (red) vs. wt (blue) mice after primary infection and weekly OVA injections. Bars and whiskers of dot plots depict means and respective standard errors of the mean (SEM). *P* values were calculated using unpaired student’s *t* test or one-way ANOVA with post-hoc Tukey’s multiple comparisons method if two or more groups were analyzed. All presented datasets are representative of at least three individual experiments with *n* ≥ 3 mice/group. Normalized MFIs were calculated by dividing the MFIs of each sample by the mean MFI of the respective wt group

Although systemic T memory formation of autoreactive OT-1 cells was limited in NOH mice, renal infiltrations persisted and even increased after booster infection. The previously identified autoreactive T memory subpopulation in NOH mice (cluster 7) may have initially fed renal infiltrations. However, these cells were probably not the only factor promoting sustained renal autoimmunity. Especially, since splenic autoreactive OT-1 cells exhibited features of T cell dysfunction (Fig. 1K, L). To explore the phenotype of kidney-specific T cells and distinguish them from their circulating counterparts, flow cytometry analyses of kidney single-cell suspensions were performed. In line with histological findings, more OT-1 cells were found in single-cell suspensions of NOH vs. wt kidneys. OT-1 frequencies further increased after booster infection (19.4 vs. 9.8% of single cells, P = 0.0180; Fig. 5A and Supplementary Fig. S12A).

Unsupervised clustering of flow cytometry data revealed a specific OT-1 subpopulation in NOH mice, expressing the tissue-resident T_RM_ cell markers CD103 and CD69, but also higher levels of the inhibitory receptors PD-1, lymphocyte activating gene-3 (LAG3) and CD39 (Fig. 5B). The majority of OT-1 cells derived from NOH mice expressed CD69, while a smaller subpopulation also expressed CD103 (CD69^+^/CD103^+^: 13.5 vs. 0.5%, *P* < 0.0001; Fig. 5C). Additional histological analysis, focusing on CD103 as a surrogate marker, revealed that these potential T_RM_ cells resided within the periglomerular and peritubular infiltrates in kidneys of NOH mice (Fig. 5D). Expression of T_RM_ makers increased over time. After primary infection, expression of CD69 and CD103 in NOH mice was lower compared to the later time point (Fig. 5C and Supplementary Fig. S12B). Hence, CD69 expression is most likely induced in the context of T_RM_ differentiation or persistent antigen recognition, and not in the context of early activation. This was further corroborated by comparable expression patterns of activation and memory markers in NOH and wt-derived renal OT-1 cells at both time points (Supplementary Fig. S12C–E, J–L). Interestingly, potential T_RM_ differentiation in the auto-immune environment appeared to correlate with increasing expression of inhibitory receptors over time (Supplementary Fig. S12F–I, M–O). Highest expression levels of PD-1 were found in CD69^+^CD103^+^ double-positive cells, with gradually lower expression of PD-1 in CD69^+^ single positive and CD69^−^CD103^−^ double negative OT-1 cells in the kidney (normalized MFI 3.9 vs. 3.0 vs. 1.0, global *P* = 0.0075; Fig. 5E). The inhibitory receptors LAG3 and CD39 were found to have comparable expression patterns (Supplementary Fig. S12P, Q). Additionally, PD-L1 expression was significantly upregulated in PTECs of infiltrated areas (Fig. 5F), which was in line with prior data showing inducible PD-L1 upregulation in renal tubular epithelial cells under inflammatory conditions [31].

To further investigate whether site-specific renal immunity and memory formation are dependent on a systemic immune response, instead of booster infection, repetitive ovalbumin injections were administered. As demonstrated previously, injected ovalbumin is quickly filtrated and presented in the renal interstitium. Hence, a cohort of mice was injected weekly with 50 µg of ovalbumin beginning on day 7 after primary LM-OVA infection. NOH mice developed a renal phenotype with infiltrations and significant proteinuria comparable to NOH mice after prime-boost infection (Fig. 5G, H). After 35 days, a high frequency of renal OT-1 cells in NOH vs. wt mice also expressed CD69 and CD103 (66.8 vs. 8.0%, *P* = 0.0007), suggesting local T_RM_ differentiation (Fig. 5I). However, antigen exposure without systemic infection was not sufficient to maintain peripheral OT-1 populations, and no transferred cells were detected in peripheral blood of NOH mice after 5 weeks (Fig. 5J).

In summary, we demonstrated that local autoantigen exposure induced differentiation of CD69^+^ and CD103^+^ autoreactive T cells in the kidney, which would be in line with adopting a T_RM_ phenotype. These T cells were likely maintained locally by continuous on-site autoantigen cross-presentation, as the systemic immune response meanwhile gradually diminished. An upregulation of the PD-1/PD-L1 axis indicated additional regulatory influences. Consequently, these potential T_RM_ cells supposedly played a pivotal role in mediating and controlling of the persistent self-reactive immune response.

### Human forms of interstitial nephritis are also driven by potential T_RM_ cells

Different forms of auto-immune interstitial nephritis in humans are characterized by infiltrates of various inflammatory cells. Renal pathology of these entities is mainly driven by cell-mediated, rather than humoral mechanisms [4, 32]. To investigate if our study model might serve as a blueprint for human forms of interstitial nephritis, we performed histopathological analysis of renal biopsies of four patients diagnosed with AIN and one patient diagnosed with ICI-associated nephritis.

**Fig. 6:**
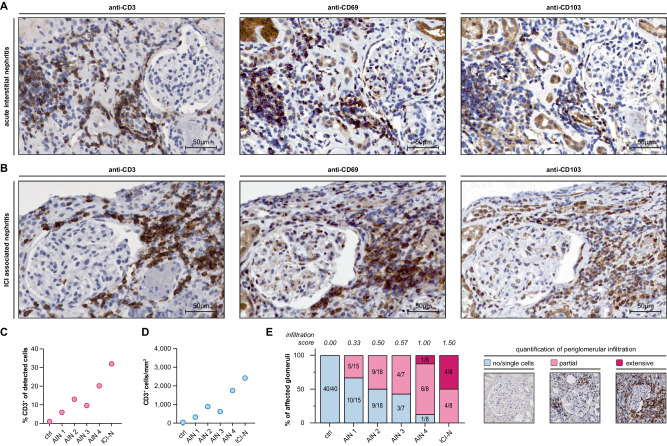
Potential T_RM_ differentiation can be detected in human forms of interstitial nephritis, with periglomerular infiltrates closely resembling those in the NOH model. **A** Representative immunohistochemistry from sequential kidney sections from a human patient diagnosed with acute interstitial nephritis (AIN), showing periglomerular T cell (CD3^+^) infiltrates (left), as well as CD69 positive (central) and CD103 positive cells (right) within the infiltrate. **B** Representative immunohistochemistry from sequential kidney sections of a human patient diagnosed with immune-checkpoint inhibitor (ICI)-associated nephritis, showing periglomerular T cell (CD3^+^) infiltrates (left), as well as CD69 positive (central) and CD103 positive cells (right) within the infiltrate. **C** Dot plot illustrating T cell percentages of all renal cells/slide in a series of four cases with AIN and one case ICI-associated nephrites. **D** Dot plot illustrating the number of T cells/mm^2^ in a series of four cases with AIN and one case ICI-associated nephrites. **E** Bar graph illustrating percentages of glomeruli, affected by periglomerular T cell infiltration. Colors depict the severity of periglomerular infiltrates in three categories. Panel on the right shows examples of the visual grading. Numbers in bars indicate fractions of glomeruli/category. An infiltration score is calculated by the following formula: IS = *c*1 × 0 + *c*2 × 1 + *c*3 × 2 (*c*1: no/single-cell fraction, *c*2: partially infiltrated fraction, *c*3: extensively infiltrated fraction of glomeruli). Ctrl healthy control

Besides interstitial infiltrates, T cell-rich periglomerular infiltrations were detected in all analyzed samples. As expected, T cell infiltrates were composed of both CD8^+^ CTLs and CD4^+^ T helper cells, while only single B cells were detectable (Supplementary Fig. S13). Interestingly, relevant subpopulations of T cells also expressed the T_RM_ associated markers CD69 and/or CD103 in both AIN and ICI-associated nephritis (Fig. 6A, B). According to the acuity of the disease, frequencies and densities of renal T cell infiltrations exhibited interindividual variations, but were present in all cases (Fig. 6C, D). The percentage of affected glomeruli and the severity of the periglomerular infiltrations were associated with overall T cell density (Fig. 6E). Of note, the most severe renal infiltration was found in ICI-associated nephritis, which could be attributed to the ICI-induced reactivation of multiple inhibited T cell clones maintaining an immune response against a diverse set of autoantigens.

Collectively, cellular infiltrates of human auto-immune interstitial nephritis closely resemble those found in the NOH model, while potential T_RM_ differentiation, as indicated by CD69 and CD103 expression, appeared to be a relevant feature of human auto-immune nephritis as well.

## Discussion

Here, we developed and harnessed a murine model of CD8 T cell-dependent cellular auto-immune nephritis to elucidate the activation and maintenance of tissue-resident autoreactive CTLs.

Intriguingly, autoreactive CD8 T cells that become activated in the presence of their cognate antigen during the acute phase of an infection develop a hybrid phenotype with features of effector development, memory formation, and dysfunction. This eventually leads to their demise in the systemic circulation. However, in a case of split tolerance, local autoantigen cross-presentation in the kidney maintains potential T_RM_ cells, where they cause auto-immune nephritis, closely resembling human forms of interstitial nephritis.

Neither adoptive transfer of naïve OT-1 CTLs nor infection with OVA-expressing pathogens could directly trigger renal autoimmunity in NOH mice, which implicates robust peripheral and central tolerance against OVA in NOH mice, respectively [33]. Yet, through combination of adoptive transfer and prime-boost infection, we were able to mount a long-lasting immune response against a tissue-specific antigen [34].

OT-1 cells served in our model as surrogates for CD8 T cells that escape negative selection and usually undergo peripheral tolerance. In vivo activation was therefore essential for the survival of transferred autoreactive OT-1 cells. After an initially pronounced effector response, an accelerated attrition of autoreactive CTLs could be observed in NOH mice. This may be explained by overstimulation and activation-induced cell death through strong TCR engagement during acute infection and concomitant autoantigen cross-presentation. Additionally, effector subpopulations exhibited functional impairment, as cytokine production (e.g., IFN-γ, TNF) was limited in peripheral OT-1 cells of NOH mice. Supporting this, higher fractions of KLRG1^+^CX3CR1^high^ OT-1 effectors were detected in NOH compared to wt mice. Our data are supported by a recent study which directly coupled CX3CR1 expression to the differentiation continuum of CTLs and demonstrated that terminally differentiated CTLs lose their ability to efficiently produce IFN-γ and TNF [35].

Corroborating our differential flow cytometry data, transcriptomic analysis revealed a hybrid T memory phenotype in OT-1 cells from NOH mice, which implies that tolerance and effector differentiation can occur within the same cell concurrently. The ultimate outcome of such a competition is most likely decided by the strength of the individual signals as shown before [36–38]. The hybrid phenotype was characterized by higher expression of “T cell exhaustion” patterns and other markers associated with T cell dysfunction (e.g., *Pdcd1*, *Ctla4*, *Lag3*, *Cd200*, *Ikzf2* [*Helios*], and *Tox*). *Tox*, for example, orchestrates early transcriptional and epigenetic programs driving fate commitment toward T cell exhaustion [39]. An aberrant T memory fate can compromise maintenance of a long-term auto-immune response. Limited memory formation was further corroborated by lower fractions of peripheral CD127^+^CX3CR1^low^ memory (precursor) CTLs and higher expression of inhibitory receptors (e.g., PD-1, Tim3) in NOH-derived OT-1 cells. Interestingly, the divergent T cell phenotype in NOH mice could already be detected during the acute phase of infection (day 7) with a stable phenotype over time. This higher expression of inhibitory receptors in combination with downregulation of the TCR in OT-1 cells from NOH mice might also explain the observed regression of functional avidity, which is in contrast to functional avidity maturation of canonical CD8 memory T cells [40, 41]. Hence, we postulate that the memory formation of autoreactive CTLs is controlled by a distinct differentiation program that diverts canonical T effector and T memory cell differentiation by partly adopting genetic programs also acting during CD8 T cell exhaustion. In line with our data, “exhaustion-like” programs have previously been linked to autoreactive CTLs (e.g., in models of diabetes mellitus and lupus nephritis) [42, 43].

Taken together, an accelerated T effector differentiation and impaired T memory cell formation can control immunity and may be crucial in mitigating a potentially overwhelming self-targeted immune response. Harnessing mechanisms that tip the balance in favor of a tolerogenic immune response could therefore be of great potential in treating immune-mediated diseases.

While peripheral OT-1 counts rapidly declined in NOH mice, significant proteinuria persisted and a robust accumulation of autoreactive cells in kidneys of NOH mice, forming dense periglomerular and peritubular infiltrates, could be detected. Interestingly, infiltrates were not just composed of transferred autoreactive CTLs, but consisted of different immune cell types (e.g., significant amounts of CD4^+^ T helper cells, F4/80^+^ monocytes/macrophages, and CD11c^+^ dendritic cells) required for a complex local immune response. To recruit autoreactive CTLs, as well as establish and maintain these intrarenal infiltrates, local cross-presentation of autoantigen is mandatory, as demonstrated by their absence in wt mice. We were able to show that antigens located within Bowman’s capsule (either filtered or shed from glomerular cells), can be effectively internalized by PTECs and eventually transported to antigen-presenting cells (APCs, e.g., F4/80^+^ monocytes/macrophages or CD11c^+^ dendritic cells) located in the periglomerular space. Here, soluble antigen could be taken up via mannose receptor-mediated endocytosis and cross-presented by APCs to initiate or maintain a CTL response [44, 45]. Recent studies could also demonstrate that PTECs can act as non-professional APCs, after internalizing soluble antigen [45]. This may be an additional mechanism contributing to intrarenal antigen cross-presentation and tubulitis. The relevance of CTLs in immune-mediated renal disease is further emphasized by their presence and correlation with disease activity in forms of human auto-immune nephritis (e.g., lupus nephritis) [46]. While it is conceivable that autoantibodies could also contribute to the pathology in our model, the low levels of OVA-specific immunoglobulins suggest that the humoral immune response has, at most, a modifying effect.

We further characterized renal infiltrations and successfully identified distinct subpopulations of renal CTLs expressing canonical tissue residency markers like CD69 and CD103. T_RM_ cells can act as vigilant immune sentinels in the tissue and play a crucial role in localized immune responses by providing inflammatory cytokines and cellular cytotoxicity. Thus, T_RM_ cells can actively contribute to local pathology [47]. Importantly, T_RM_ cells have the ability to self-renew, forming a specialized tissue-specific T memory population. Recent studies have suggested a role for autoreactive T_RM_ cells in the context of various auto-immune diseases by contributing to long-lasting tissue damage. In mouse models of lupus, for example, kidney-specific T_RM_ cells were identified, and their prevalence was found to positively correlate with disease activity [48, 49].

While detailed functions of T_RM_ cells in the context of auto-immune nephritis are still elusive, our data strongly indicate that self-renewing T_RM_ cells might promote persistent local autoimmunity. We could detect potential T_RM_ cells as early as day 7 in our nephritis model and differentiation of CD69^+^ and/or CD103^+^ positive cells was dependent on local autoantigen dose, as higher fractions differentiated in kidneys of NOH mice that were administered weekly ovalbumin injections instead of a booster infection. Notably, no significant numbers of circulating OT-1 cells were left in these mice after 35 days. Hence, autonomous renal T_RM_ cell populations might be the key drivers of a self-targeted local immune response, without requiring continuous replenishment from the periphery. However, it remains uncertain whether potential T_RM_ cells differentiate locally from infiltrating CTLs or if distinct circulating precursor cells, poised for T_RM_ differentiation, populate the kidneys during the initial phases of inflammation [50]. T_RM_ differentiation has been associated with higher expression of, e.g., *Znf683*, *Itgae*, *Cd69*, *Xcl1*, *Ahr*, *Nr4a1*, *Runx3*, *Egr2*, *Prdm1*, and *P2rx7* as well as reduced expression of *Klf2* and *S1pr1* [51–56]. Of note, we found circulating OT-1 cells at day 7 (cluster 7) from NOH mice that showed higher expression of *Itgae*, *Cd69*, *Xcl1*, *Ahr*, *Nr4a1*, *Egr2*, and *P2rx7*, while *Klf2* and *S1pr1* were concomitantly downregulated. Thus, a circulating subpopulation of T_RM_ precursors might have its origin within cluster 7. These data promote further studies addressing the origin and replenishment of kidney-specific T_RM_ cells.

Furthermore, T cells residing in the kidneys of NOH mice, like their potential peripheral precursor population, exhibited a moderate upregulation of inhibitory receptors such as PD-1, Tim-3, LAG3, and CD39, with the highest levels observed in CD69^+^CD103^+^ double-positive cells. Contribution of inhibitory signaling to regulating and fine-tuning of the renal immune response was further corroborated by enhanced PD-L1 expression in PTECs and other cell types within the infiltrated areas. Heightened signaling through inhibitory receptors might also lower the functional avidity of OT-1 T cells, which may serve as negative feedback to limit tissue damage. The occurrence of primary cell-mediated auto-immune nephritis in patients treated with checkpoint inhibitors (e.g., anti-PD-1 or anti-CTLA-4 monoclonal antibodies) further strengthens the hypothesis that inhibitory signals are necessary to control self-reactive tissue-resident T cells. Inhibitory molecules have also been found on murine and human T_RM_ cells from various locations and additionally support the concept of a self-regulating immune response within the tissue niche [57–60].

A translational aspect of our model is strongly supported by the identification of comparable CD69^+^ and CD103^+^ T cell subpopulations in kidney samples from patients with various types of auto-immune nephritis, including AIN or ICI-related nephritis. In human kidneys, the infiltrations closely resembled those observed in NOH mice in terms of immune cell density and localization. Thus, our data contribute to the expanding body of evidence suggesting that T_RM_ cells likely play a pivotal role not just in the context of host defense but also in promoting autoimmunity. Inhibition of autoreactive T cells within the tissue niche could therefore be a viable therapeutic approach for treating auto-immune nephritis. Based on our data, augmentation of inhibitory signaling (e.g., Fc-fused PD-L1), in T_RM_ cells might be a suitable therapeutic strategy in already established disease [61, 62]. As a limitation of our study, it should be acknowledged that the NOH model only partially replicates (acute) interstitial nephritis. Although OVA expression on podocytes suggests the possibility of additional intraglomerular antigen recognition, our findings demonstrate effective OVA transport through PTECs eliciting the local immune response in the tubulointerstitium.

In summary, our study yields profound insights into the dynamics of cell-driven renal autoimmunity. We have demonstrated that autoreactive CTLs can attain activation, temporarily bypassing peripheral self-tolerance when exposed to pathogens expressing their cognate antigens. Furthermore, we have highlighted the process by which antigen passing through Bowman’s capsule reaches the periglomerular space, where it is cross-presented and eventually activates autoreactive CTLs. While the systemic immune response fades rapidly in the autoreactive context, a robust renal response persists through local autoantigen cross-presentation. Within the kidneys, we were able to identify a specialized subset of autoreactive CD69^+^CD103^+^ T cells that bears canonical hallmarks of T_RM_ cells and likely plays a pivotal role in sustaining long-lasting local autoimmunity. Lastly, we also detected potential T_RM_ cell subsets in kidney sections from patients with auto-immune nephritis, suggesting the potential of our model to develop innovative therapeutic approaches to treat cell-driven autoimmunity. Taken together, our findings significantly complement current knowledge about function and regulation of self-targeted cellular immunity and pathogenesis of auto-immune nephritis. Eventually, they might help to identify potential therapeutic targets for the treatment of CTL-driven auto-immune nephritis.

## Materials and methods

### Study design

In this study, we developed a murine model of cellular auto-immune nephritis to investigate the differentiation and functions of self-targeted CD8^+^ T cells systemically and locally. To establish renal autoimmunity, we used NOH (nephrin, ovalbumin, HEL) mice, expressing the neo-autoantigen ovalbumin on the surface of podocytes (Supplementary Fig. S1A, B) [14]. Autoreactive CD90.1^+^OT-1 cells (referred to as OT-1 cells) with high avidity for MHC class I restricted ovalbumin were adoptively transferred into wt and NOH hosts. Transferred OT-1 cells could be traced via anti-CD90.1 staining in hosts on C57BL/6 (CD90.2) background. In vivo, activation of OT-1 cells was accomplished via primary infection on day 1 after adoptive transfer using recombinant *Listeria monocytogenes* that express the neo-antigen ovalbumin (LM-OVA) [17, 63]. To maintain high levels of autoreactive OT-1 cells, secondary heterologous booster infection with recombinant vesicular stomatitis virus, encoding ovalbumin (VSV-OVA) was performed on day 21 [18]. Both pathogens induce a predictable and well-characterized cytotoxic immune response and robust CD8^+^ T memory differentiation [64, 65]. Weekly blood and urine analyses were performed to track CD8 T cell responses and kidney damage. Mice were euthanized for final analysis on day 7 and days 35–57 (Fig. 1A). To unravel renal antigen presentation wt mice were i.v. injected with labeled ovalbumin and euthanized for histopathological and FACS analysis, 30, 60, and 120 min after injection. Experimental groups consisted of 3–5 mice and each experiment was replicated at least three times.

### Mice

All mice were maintained in specific-pathogen-free facilities at the Institute of Microbiology and Hygiene, Medical Center – University of Freiburg, Germany. Mice were provided a standardized environment with filtered air, controlled temperature, and humidity, a 12-h light–dark cycle, and unlimited access to water and standardized food. C57BL/6 mice were purchased from Janvier Laboratories (LeGenest St-Isle, France) and are referred to as wt mice throughout the manuscript. NOH transgenic (C57BL/6-Tg(NOH)) founder mice were kindly provided by the Kurts lab (Institute of Molecular Medicine & Experimental Immunology, University of Bonn, Germany) and bred locally [14]. OT-1 transgenic (C57BL/6-Tg(TcraTcrb)1100^Mjb^) mice were maintained locally on a Thy1.1 congenic (B6.PL-Thy1^a^/CyJ) background to obtain CD90.1^+^ OT-1 lymphocytes for transfer [15]. For all transfer experiments, donor and recipient mice were sex-matched. All experimental cohorts were sex and age matched and all mice were between 6 and 16 weeks of age at the start of the experiments. All animal experiments were approved and conducted according to Institutional and National Guidelines (Approval Number G21/021, Regional Council Freiburg i. Br., Germany).

### Adoptive CD8^+^ T cell transfer

Donor CD90.1^+^ OT-1 cells were isolated from the spleens and lymph nodes of euthanized mice. Single-cell suspensions were generated by mechanical disruption under sterile conditions. After red cell lysis, frequencies of CD8α^+^CD90.1^+^TCRVα1^+^TCR-Vβ5.1^+^ lymphocytes (OT-1 cells) were obtained via flow cytometry and multiplied with the manual count of living lymphocytes. The concentration of single-cell suspensions was adjusted to 10^7^ OT-1 cells/ml and each host mouse was i.v. injected with 200 µl (2 × 10^6^ OT-1 cells) of single-cell suspension. For each experiment, OT-1 cells of 2–3 pooled donor mice were transferred in cohorts of 10 mice (5 NOH and 5 wt mice). For the in vivo CFSE assay, cells were labeled prior to transfer, using a CFSE Cell Division Tracker Kit (BioLegend) according to the manufacturer’s protocol.

### Infection and ovalbumin injection

Recombinant LM-OVA was acquired from log phase of growth in BHI medium. Each mouse was i.v. infected with 5000 cfu LM-OVA 24 h after OT-1 transfer. The final suspensions were plated out to post-hoc check the concentration used for infection. For booster infection, each mouse was i.v. injected with 2 × 10^6^ pfu VSV-OVA on day 21. VSV-OVA stocks were kindly provided by the Pinschewer Lab (Department of Biomedicine, University of Basel, Switzerland).

For the renal cross-presentation experiments, mice were i.v. injected with 50 µg of fluorophore-labeled ovalbumin (Alexa Fluor 488-OVA or Alexa Fluor 555-OVA) 30, 60, or 120 min prior to sacrifice.

### Cell isolation from tissues

Lymphocytes from spleens and lymph nodes were isolated via mechanical disruption by passing the tissue through a 70 µm cell strainer, followed by red cell lysis and resuspension in phosphate-buffered saline (PBS). Cell concentrations were adjusted according to manual counts of living lymphocytes. For the preparation of single-cell suspensions from the kidneys, organs were minced, pre-digested with collagenase (at 37 °C for 60 min), mechanically disrupted, and finally filtered through a 70 µm cell strainer. Lymphocytes were isolated using density gradient centrifugation by resuspending the cell pellets in 5 ml of 40% Percoll in RPMI layered onto 5 ml of 80% Percoll in PRPMI and centrifugation for 20 min at 400 × *g*. Concentrated leukocytes and remaining tissue epithelial cells were collected from the interphase, washed, resuspended in PBS, and adjusted to final concentrations according to manual counts of living lymphocytes [54]. Peripheral blood was analyzed after red cell lysis.

### Flow cytometry

Single-cell suspensions of tissues were labeled for 30–60 min on ice, using the antibodies listed in Supplementary Table S1. In vitro, restimulation was performed by incubating 5 × 10^6^ cells in 200 µl RPMI medium with different concentrations of SIINFEKL peptide (2 × 10^−14^–2 × 10^−^^6^ M) and 20 µg/ml brefeldin A over 4 h at 37 °C. After washing and surface staining, cells were fixated using BD Cytofix fixation buffer (Ref. 554655, BD Biosciences, Franklin Lakes, NJ, USA) according to the manufacturer’s specifications. Staining for intracellularly accumulated cytokines was performed in permeabilization buffer (0.5% saponin in PBS) using the antibodies listed in Supplementary Table S2. Samples were acquired on an LSRFortessa flow cytometer (BD Biosciences, Franklin Lakes, NJ, USA). Flow cytometry data were analyzed using FlowJo V10.8 software (TreeStar, Ashland, OR, USA). Dimensionality reduction of flow cytometry data was performed after downsampling and concatenation, using UMAP as implemented in the UMAP plugin (Version 3.3.4) [66]. Semi-unsupervised cluster identification was achieved by generating self-organizing maps (SOM) applying the R-based plugin FlowSOM (Version 3.0.18) [67].

### Histological analysis of mouse and human samples

For immunofluorescence, spleen, lymph node, and kidney sections were fixated in 4% formaldehyde solution (2 h on ice), dehydrated in 30% sucrose solution (>12 h), embedded (Tissue-Tek O.C.T. Compound), and snap frozen in liquid nitrogen. Cryosections of 6 µm thickness were blocked for 30 min with 5% bovine serum albumin containing Fc-block (1:200) followed by incubation with mixtures of directly fluorophore-labeled antibodies and DAPI for 60 min. Used antibodies are listed in Supplementary Table S2. After mounting and sealing, epifluorescence images were acquired with an Axioplan 2 Fluorescence Microscope equipped using a 20-fold and 40-fold objective. Confocal laser scanning microscopy was performed with an LSM880 Confocal Microscope (both Zeiss, Oberkochen, Germany).

For classical histology, 2 µm sections of murine formalin fixed paraffin embedded (FFPE) kidney tissue were stained with the periodic acid Schiff method (0.5% periodic acid solution, followed by Schiff’s reagent, counterstained with hematoxylin). Slides were scanned using a slide scanner (VENTANA DP 200, Roche Diagnostics, Basel, Switzerland).

Iterative indirect immunofluorescence imaging (4i) on FFPE tissue sections of murine kidneys was performed as recently described [68]. Primary antibodies used for labelling are listed in Supplementary Table S3. Images were acquired using an inverted Zeiss Axio Observer microscope equipped with a scanning stage and 20-fold objective (Zeiss, Oberkochen, Germany).

For immunohistochemistry analysis of human kidney biopsy samples, 2 µm FFPE sections were stained using the antibodies listed in Supplementary Table S4. Automated staining was performed with Agilent antibodies using the Autostainer Link 48 (Agilent Dako, Santa Clara, CA, USA) according to the manufacturer’s protocol. Additional manual staining procedures, including heat-induced antigen retrieval (citrate pH6) for CD103 and DAB staining, were performed as recently described [68]. Slides were scanned using a slide scanner (VENTANA DP 200). To reduce variations between the different stainings, automated color deconvolution and normalization were performed on the RGB images using ImageJ (Version 1.53s) [69]. The local Ethics Committee of the Medical Center, University of Freiburg approved the use of human cancer nephrectomy (control) and kidney biopsy (AIN) samples (EK 21/1288 and EK 512/18). Informed consent was obtained from all individual participants included in the study. The study is in conformity with the Helsinki and Istanbul declarations and their amendments.

### Semi-automated cell quantification in kidney sections

Cell type detection and quantification of whole kidney immunofluorescence sections and 4i-stainings were performed using QuPath (Version 0.3.2) [70].

### Single-cell RNA-sequencing analysis

Single-cell suspensions of spleen and lymph nodes of two wt and two NOH mice at day 7 after infection were pre-enriched for CD8^+^ cells applying negative selection immunomagnetic cell separation using the Dynabeads™ Untouched™ Mouse CD8 Cells Kit (Invitrogen, Waltham, MA, USA) as per manufacturer’s protocol, except for the addition of CD90.2 antibody (1:100) to the stock antibody mix to increase selection purity. CD8α^+^CD90.1^+^DAPI^−^ OT-1 cells were isolated from the pre-enriched cell suspensions by flow cytometry-assisted cell sorting using an FACSAria III (BD Biosciences, Franklin Lakes, NJ) cytometer. Sorted OT-1 cells of each sample were labeled with different oligonucleotide-tagged TotalSeq anti-CD45/anti-MHCI hashing antibodies as listed in Supplementary Table S5. Hashed cells were multiplexed and loaded on a GemCode Single-Cell Instrument (10x Genomics, Pleasanton, CA, USA) to generate single-cell gel beads in emulsion. Gene expression and feature barcode libraries were generated as described in the user’s guide for 10x Chromium Single Cell Reagent Kits with feature barcoding technology for cell surface protein. The library pool was commercially sequenced on an Illumina NovaSeq6000 platform (PE150 bp) at Novogene Europe (Cambridge, UK). Demultiplexed FASTQ files were generated from raw sequencing data. Cell Ranger (Version 6.0.0, 10x Genomics) software was used to perform alignment against the murine reference genome and gene annotation (Cell Ranger reference package mm10-2020-A based on GRCm38/mm10 and Genocode Release vM23), barcode processing, and gene counting. Downstream bioinformatics analysis was performed using the R package Seurat (Version 4.3.0) [71]. Samples were demultiplexed based on the enrichment of hashtag oligos (HTOs) applying the HTODemux function integrated in Seurat. The demuxed feature/barcode matrices were filtered for cells with a feature counts >1500 and a percentage of mitochondrial genes <5%. Mitochondrial, ribosomal, and unknown features were excluded from further analysis. The filtered matrix was log-normalized for the library size of each cell. Gene expression levels of the 2000 most highly variable features were scaled and dimensionality reduction was performed on these genes using principal component analysis. Clusters were identified via the graph-based clustering approach implemented in the FindNeighbors and FindClusters functions of Seurat (dimensionality of the dataset was estimated to be 30, resolution parameter was set to 0.8). A two-dimensional embedding of cells from significant principal components was generated using UMAP. Gene ontology (GO) term enrichment analyses were performed using gene sets derived from the mouse GO biological process (BP) ontology database. Additional GSEA was performed using the MSigDB “exhausted vs. memory CD8 T cell” datasets (accession number GSE9650) [72]. For enrichment analysis and visualization, the clusterProfiler and enrichplot packages were used [73]. Pseudotemporal ordering for the construction of single-cell trajectories was done with monocle3 [74–76].

### Statistical analysis of flow cytometry and histological data

Statistical analysis was performed using Prism Version 9.3.1 (GraphPad Software, Boston, MA, USA). Data were compared applying unpaired Student’s *t* tests or one-way analysis of variance (ANOVA) with post-hoc Tukey’s multiple comparison, if more than two groups were compared. Parametric tests were performed under the assumption of normally distributed data, which was not formally tested. All data points are individually shown in the graphs with corresponding mean and standard error of the mean (SEM) depicted by horizontal bars and whiskers. Exact *P* values are reported in the figures. Two-tailed *P* values < 0.05 were considered statistically significant.

### Supplementary information

Supplementary Material

Raw Data File

## Data Availability

All data are available in the main text or the Supplementary Materials. Single-cell RNA-seq data generated in this manuscript are available from the Gene Expression Omnibus (GEO) under Accession Number GSE249212. The following publicly available datasets were used for analysis: mouse genome for alignment of sequencing data: pre-built Cell Ranger mouse reference 2020-A (July 7, 2020); effector vs. memory CD8 T cell gene sets available under the GEO accession number GSE9650. Curated gene sets for circulating vs. resident and activated vs. exhausted T cells as published by Crowl et al. [54].

## Code availability

Standard R packages were used for data analysis and generation of figures as describes in the “Materials and Methods” section. Specific computer codes are available upon reasonable request from the corresponding author.

## References

[1] Kant S, Kronbichler A, Sharma P, Geetha D. Advances in understanding of pathogenesis and treatment of immune-mediated kidney disease: a review. Am J Kidney Dis. 2022;79:582–600. 34508831 10.1053/j.ajkd.2021.07.019

[2] Kurts C, Klebba I, Davey GM, Koch KM, Miller JF, Heath WR, et al. Kidney protection against autoreactive CD8(+) T cells distinct from immunoprivilege and sequestration. Kidney Int. 2001;60:664–71. 11473649 10.1046/j.1523-1755.2001.060002664.x

[3] Linke A, Tiegs G, Neumann K. Pathogenic T-cell responses in immune-mediated glomerulonephritis. Cells. 2022;11:1625. 10.3390/cells11101625 PMC9139939 35626662

[4] Praga M, Gonzalez E. Acute interstitial nephritis. Kidney Int. 2010;77:956–61. 20336051 10.1038/ki.2010.89

[5] Baker RJ, Pusey CD. The changing profile of acute tubulointerstitial nephritis. Nephrol Dial Transplant. 2004;19:8–11. 14671029 10.1093/ndt/gfg464

[6] Sprangers B, Leaf DE, Porta C, Soler MJ, Perazella MA. Diagnosis and management of immune checkpoint inhibitor-associated acute kidney injury. Nat Rev Nephrol. 2022;18:794–805. 36168055 10.1038/s41581-022-00630-8

[7] Casey KA, Fraser KA, Schenkel JM, Moran A, Abt MC, Beura LK, et al. Antigen-independent differentiation and maintenance of effector-like resident memory T cells in tissues. J Immunol. 2012;188:4866–75. 22504644 10.4049/jimmunol.1200402 PMC3345065

[8] Jiang X, Clark RA, Liu L, Wagers AJ, Fuhlbrigge RC, Kupper TS. Skin infection generates non-migratory memory CD8+ T(RM) cells providing global skin immunity. Nature. 2012;483:227–31. 22388819 10.1038/nature10851 PMC3437663

[9] Li L, Tang W, Zhang Y, Jia M, Wang L, Li Q, et al. Targeting tissue-resident memory CD8(+) T cells in the kidney is a potential therapeutic strategy to ameliorate podocyte injury and glomerulosclerosis. Mol Ther. 2022;30:2746–59. 35514086 10.1016/j.ymthe.2022.04.024 PMC9372318

[10] McGaha TL, Madaio MP. Lupus nephritis: animal modeling of a complex disease syndrome pathology. Drug Discov Today Dis Models. 2014;11:13–8. 25722732 10.1016/j.ddmod.2014.08.002 PMC4337231

[11] Shochet L, Holdsworth S, Kitching AR. Animal models of ANCA associated vasculitis. Front Immunol. 2020;11:525. 32373109 10.3389/fimmu.2020.00525 PMC7179669

[12] Imai H, Nakamoto Y, Asakura K, Miki K, Yasuda T, Miura AB. Spontaneous glomerular IgA deposition in ddY mice: an animal model of IgA nephritis. Kidney Int. 1985;27:756–61. 4021309 10.1038/ki.1985.76

[13] Ougaard MKE, Kvist PH, Jensen HE, Hess C, Rune I, Søndergaard H. Murine nephrotoxic nephritis as a model of chronic kidney disease. Int J Nephrol. 2018;2018:8424502. 29692933 10.1155/2018/8424502 PMC5859794

[14] Heymann F, Meyer-Schwesinger C, Hamilton-Williams EE, Hammerich L, Panzer U, Kaden S, et al. Kidney dendritic cell activation is required for progression of renal disease in a mouse model of glomerular injury. J Clin Investig. 2009;119:1286–97. 19381017 10.1172/JCI38399 PMC2673875

[15] Hogquist KA, Jameson SC, Heath WR, Howard JL, Bevan MJ, Carbone FR. T cell receptor antagonist peptides induce positive selection. Cell. 1994;76:17–27. 8287475 10.1016/0092-8674(94)90169-4

[16] Pape KA, Kearney ER, Khoruts A, Mondino A, Merica R, Chen ZM, et al. Use of adoptive transfer of T-cell-antigen-receptor-transgenic T cell for the study of T-cell activation in vivo. Immunol Rev. 1997;156:67–78. 9176700 10.1111/j.1600-065X.1997.tb00959.x

[17] Pope C, Kim SK, Marzo A, Masopust D, Williams K, Jiang J, et al. Organ-specific regulation of the CD8 T cell response to *Listeria monocytogenes* infection. J Immunol. 2001;166:3402–9. 11207297 10.4049/jimmunol.166.5.3402

[18] Kim SK, Reed DS, Olson S, Schnell MJ, Rose JK, Morton PA, et al. Generation of mucosal cytotoxic T cells against soluble protein by tissue-specific environmental and costimulatory signals. Proc Natl Acad Sci USA. 1998;95:10814–9. 9724787 10.1073/pnas.95.18.10814 PMC27978

[19] Nelson CE, Thompson EA, Quarnstrom CF, Fraser KA, Seelig DM, Bhela S, et al. Robust iterative stimulation with self-antigens overcomes CD8(+) T cell tolerance to self- and tumor antigens. Cell Rep. 2019;28:3092–104.e5. 31533033 10.1016/j.celrep.2019.08.038 PMC6874401

[20] Dubois PM, Pihlgren M, Tomkowiak M, Van Mechelen M, Marvel J. Tolerant CD8 T cells induced by multiple injections of peptide antigen show impaired TCR signaling and altered proliferative responses in vitro and in vivo. J Immunol. 1998;161:5260–7. 9820498 10.4049/jimmunol.161.10.5260

[21] Chang JT, Wherry EJ, Goldrath AW. Molecular regulation of effector and memory T cell differentiation. Nat Immunol. 2014;15:1104–15. 25396352 10.1038/ni.3031 PMC4386685

[22] Renkema KR, Huggins MA, Borges da Silva H, Knutson TP, Henzler CM, Hamilton SE. KLRG1(+) memory CD8 T cells combine properties of short-lived effectors and long-lived memory. J Immunol. 2020;205:1059–69. 32611727 10.4049/jimmunol.1901512 PMC7415731

[23] Jung YW, Rutishauser RL, Joshi NS, Haberman AM, Kaech SM. Differential localization of effector and memory CD8 T cell subsets in lymphoid organs during acute viral infection. J Immunol. 2010;185:5315–25. 20921525 10.4049/jimmunol.1001948 PMC4267692

[24] Martin MD, Badovinac VP. Defining memory CD8 T cell. Front Immunol. 2018;9:2692. 30515169 10.3389/fimmu.2018.02692 PMC6255921

[25] Zhou X, Yu S, Zhao DM, Harty JT, Badovinac VP, Xue HH. Differentiation and persistence of memory CD8(+) T cells depend on T cell factor 1. Immunity. 2010;33:229–40. 20727791 10.1016/j.immuni.2010.08.002 PMC2928475

[26] Szabo PA, Levitin HM, Miron M, Snyder ME, Senda T, Yuan J, et al. Single-cell transcriptomics of human T cells reveals tissue and activation signatures in health and disease. Nat Commun. 2019;10:4706. 31624246 10.1038/s41467-019-12464-3 PMC6797728

[27] McLane LM, Abdel-Hakeem MS, Wherry EJ. CD8 T cell exhaustion during chronic viral infection and cancer. Annu Rev Immunol. 2019;37:457–95. 30676822 10.1146/annurev-immunol-041015-055318

[28] Utzschneider DT, Gabriel SS, Chisanga D, Gloury R, Gubser PM, Vasanthakumar A, et al. Early precursor T cells establish and propagate T cell exhaustion in chronic infection. Nat Immunol. 2020;21:1256–66. 32839610 10.1038/s41590-020-0760-z

[29] Chen A, Lee K, D'Agati VD, Wei C, Fu J, Guan TJ, et al. Bowman’s capsule provides a protective niche for podocytes from cytotoxic CD8+ T cells. J Clin Investig. 2018;128:3413–24. 29985168 10.1172/JCI97879 PMC6063505

[30] Trzpis M, Popa ER, McLaughlin PM, van Goor H, Timmer A, Bosman GW, et al. Spatial and temporal expression patterns of the epithelial cell adhesion molecule (EpCAM/EGP-2) in developing and adult kidneys. Nephron Exp Nephrol. 2007;107:e119–31. 18025791 10.1159/000111039

[31] Schoop R, Wahl P, Le Hir M, Heemann U, Wang M, Wüthrich RP. Suppressed T-cell activation by IFN-gamma-induced expression of PD-L1 on renal tubular epithelial cells. Nephrol Dial Transplant. 2004;19:2713–20. 15353579 10.1093/ndt/gfh423

[32] Perazella MA, Shirali AC. Immune checkpoint inhibitor nephrotoxicity: what do we know and what should we do? Kidney Int. 2020;97:62–74. 31685311 10.1016/j.kint.2019.07.022

[33] Mueller DL. Mechanisms maintaining peripheral tolerance. Nat Immunol. 2010;11:21–7. 20016506 10.1038/ni.1817

[34] Rojas M, Restrepo-Jiménez P, Monsalve DM, Pacheco Y, Acosta-Ampudia Y, Ramírez-Santana C, et al. Molecular mimicry and autoimmunity. J Autoimmun. 2018;95:100–23. 30509385 10.1016/j.jaut.2018.10.012

[35] Zwijnenburg AJ, Pokharel J, Varnaitė R, Zheng W, Hoffer E, Shryki I, et al. Graded expression of the chemokine receptor CX3CR1 marks differentiation states of human and murine T cells and enables cross-species interpretation. Immunity. 2023;56:1955–74.e10. 37490909 10.1016/j.immuni.2023.06.025

[36] Vezys V, Olson S, Lefrancois L. Expression of intestine-specific antigen reveals novel pathways of CD8 T cell tolerance induction. Immunity. 2000;12:505–14. 10843383 10.1016/S1074-7613(00)80202-2

[37] Vezys V, Lefrancois L. Cutting edge: inflammatory signals drive organ-specific autoimmunity to normally cross-tolerizing endogenous antigen. J Immunol. 2002;169:6677–80. 12471097 10.4049/jimmunol.169.12.6677

[38] Nelson CE, Mills LJ, McCurtain JL, Thompson EA, Seelig DM, Bhela S, et al. Reprogramming responsiveness to checkpoint blockade in dysfunctional CD8 T cells. Proc Natl Acad Sci USA. 2019;116:2640–5. 30679280 10.1073/pnas.1810326116 PMC6377494

[39] Khan O, Giles JR, McDonald S, Manne S, Ngiow SF, Patel KP, et al. TOX transcriptionally and epigenetically programs CD8(+) T cell exhaustion. Nature. 2019;571:211–8. 31207603 10.1038/s41586-019-1325-x PMC6713202

[40] Schönrich G, Kalinke U, Momburg F, Malissen M, Schmitt-Verhulst AM, Malissen B, et al. Down-regulation of T cell receptors on self-reactive T cells as a novel mechanism for extrathymic tolerance induction. Cell. 1991;65:293–304. 1849799 10.1016/0092-8674(91)90163-S

[41] Sandu I, Cerletti D, Claassen M, Oxenius A. Exhausted CD8(+) T cells exhibit low and strongly inhibited TCR signaling during chronic LCMV infection. Nat Commun. 2020;11:4454. 32901001 10.1038/s41467-020-18256-4 PMC7479152

[42] Grebinoski S, Zhang Q, Cillo AR, Manne S, Xiao H, Brunazzi EA, et al. Autoreactive CD8(+) T cells are restrained by an exhaustion-like program that is maintained by LAG3. Nat Immunol. 2022;23:868–77. 35618829 10.1038/s41590-022-01210-5 PMC9179227

[43] Smita S, Chikina M, Shlomchik MJ, Tilstra JS. Heterogeneity and clonality of kidney-infiltrating T cells in murine lupus nephritis. JCI Insight. 2022;7:e156048. 10.1172/jci.insight.156048 PMC9089785 35271505

[44] Burgdorf S, Kautz A, Bohnert V, Knolle PA, Kurts C. Distinct pathways of antigen uptake and intracellular routing in CD4 and CD8 T cell activation. Science. 2007;316:612–6. 17463291 10.1126/science.1137971

[45] Linke A, Cicek H, Müller A, Meyer-Schwesinger C, Melderis S, Wiech T, et al. Antigen cross-presentation by murine proximal tubular epithelial cells induces cytotoxic and inflammatory CD8(+) T cells. Cells. 2022;11:1510. 10.3390/cells11091510 PMC9104549 35563816

[46] Couzi L, Merville P, Deminière C, Moreau JF, Combe C, Pellegrin JL, et al. Predominance of CD8+ T lymphocytes among periglomerular infiltrating cells and link to the prognosis of class III and class IV lupus nephritis. Arthritis Rheum. 2007;56:2362–70. 17599764 10.1002/art.22654

[47] Schenkel JM, Masopust D. Tissue-resident memory T cells. Immunity. 2014;41:886–97. 25526304 10.1016/j.immuni.2014.12.007 PMC4276131

[48] Masopust D, Soerens AG. Tissue-resident T cells and other resident leukocytes. Annu Rev Immunol. 2019;37:521–46. 30726153 10.1146/annurev-immunol-042617-053214 PMC7175802

[49] Zhou M, Guo C, Li X, Huang Y, Li M, Zhang T, et al. JAK/STAT signaling controls the fate of CD8^+^CD103^+^ tissue-resident memory T cell in lupus nephritis. J Autoimmun. 2020;109:102424–102424. 32085893 10.1016/j.jaut.2020.102424

[50] Kok L, Masopust D, Schumacher TN. The precursors of CD8^+^ tissue resident memory T cells: from lymphoid organs to infected tissues. Nat Rev Immunol. 2022;22:283–93. 34480118 10.1038/s41577-021-00590-3 PMC8415193

[51] Skon CN, Lee JY, Anderson KG, Masopust D, Hogquist KA, Jameson SC. Transcriptional downregulation of S1pr1 is required for the establishment of resident memory CD8+ T cells. Nat Immunol. 2013;14:1285–93. 24162775 10.1038/ni.2745 PMC3844557

[52] Mueller SN, Mackay LK. Tissue-resident memory T cells: local specialists in immune defence. Nat Rev Immunol. 2016;16:79–89. 26688350 10.1038/nri.2015.3

[53] Milner JJ, Goldrath AW. Transcriptional programming of tissue-resident memory CD8^+^ T cells. Curr Opin Immunol. 2018;51:162–9. 29621697 10.1016/j.coi.2018.03.017 PMC5943164

[54] Crowl JT, Heeg M, Ferry A, Milner JJ, Omilusik KD, Toma C, et al. Tissue-resident memory CD8^+^ T cells possess unique transcriptional, epigenetic and functional adaptations to different tissue environments. Nat Immunol. 2022;23:1121–31. 35761084 10.1038/s41590-022-01229-8 PMC10041538

[55] Borges da Silva H, Peng C, Wang H, Wanhainen KM, Ma C, Lopez S, et al. Sensing of ATP via the purinergic receptor P2RX7 promotes CD8^+^ Trm cell generation by enhancing their sensitivity to the cytokine TGF-beta. Immunity. 2020;53:158–71 e6. 32640257 10.1016/j.immuni.2020.06.010 PMC8026201

[56] Li G, Srinivasan S, Wang L, Ma C, Guo K, Xiao W, et al. TGF-beta-dependent lymphoid tissue residency of stem-like T cells limits response to tumor vaccine. Nat Commun. 2022;13:6043. 36229613 10.1038/s41467-022-33768-x PMC9562983

[57] Kumar BV, Ma W, Miron M, Granot T, Guyer RS, Carpenter DJ, et al. Human tissue-resident memory T cells are defined by core transcriptional and functional signatures in lymphoid and mucosal sites. Cell Rep. 2017;20:2921–34. 28930685 10.1016/j.celrep.2017.08.078 PMC5646692

[58] Dudek M, Pfister D, Donakonda S, Filpe P, Schneider A, Laschinger M, et al. Auto-aggressive CXCR6^+^ CD8 T cells cause liver immune pathology in NASH. Nature. 2021;592:444–9. 33762736 10.1038/s41586-021-03233-8

[59] Pallett LJ, Davies J, Colbeck EJ, Robertson F, Hansi N, Easom N, et al. IL-2^high^ tissue-resident T cells in the human liver: sentinels for hepatotropic infection. J Exp Med. 2017;214:1567–80. 28526759 10.1084/jem.20162115 PMC5461007

[60] Hombrink P, Helbig C, Backer RA, Piet B, Oja AE, Stark R, et al. Programs for the persistence, vigilance and control of human CD8^+^ lung-resident memory T cells. Nat Immunol. 2016;17:1467–78. 27776108 10.1038/ni.3589

[61] Song MY, Hong CP, Park SJ, Kim JH, Yang BG, Park Y, et al. Protective effects of Fc-fused PD-L1 on two different animal models of colitis. Gut. 2015;64:260–71. 24902766 10.1136/gutjnl-2014-307311

[62] Reynolds J, Sando GS, Marsh OB, Salama AD, Evans DJ, Cook HT, et al. Stimulation of the PD-1/PDL-1 T-cell co-inhibitory pathway is effective in treatment of experimental autoimmune glomerulonephritis. Nephrol Dial Transplant. 2012;27:1343–50. 21965585 10.1093/ndt/gfr529

[63] Foulds KE, Zenewicz LA, Shedlock DJ, Jiang J, Troy AE, Shen H. Cutting edge: CD4 and CD8 T cells are intrinsically different in their proliferative responses. J Immunol. 2002;168:1528–32. 11823476 10.4049/jimmunol.168.4.1528

[64] Zenewicz LA, Shen H. Innate and adaptive immune responses to *Listeria monocytogenes*: a short overview. Microbes Infect. 2007;9:1208–15. 17719259 10.1016/j.micinf.2007.05.008 PMC2042024

[65] Cobleigh MA, Bradfield C, Liu Y, Mehta A, Robek MD. The immune response to a vesicular stomatitis virus vaccine vector is independent of particulate antigen secretion and protein turnover rate. J Virol. 2012;86:4253–61. 22345454 10.1128/JVI.05991-11 PMC3318607

[66] McInnes L, Healy J, Melville J. UMAP: Uniform Manifold Approximation and Projection for dimension reduction. arXiv: 1802.03426 [Preprint]. 2018;3:861. Available from: 10.48550/arXiv.1802.03426

[67] Van Gassen S, Callebaut B, Van Helden MJ, Lambrecht BN, Demeester P, Dhaene T, et al. FlowSOM: using self-organizing maps for visualization and interpretation of cytometry data. Cytom A. 2015;87:636–45. 10.1002/cyto.a.22625 25573116

[68] Rogg M, Maier JI, Van Wymersch C, Helmstädter M, Sammarco A, Lindenmeyer M, et al. α-Parvin defines a specific integrin adhesome to maintain the glomerular filtration barrier. J Am Soc Nephrol. 2022;33:786–808. 35260418 10.1681/ASN.2021101319 PMC8970443

[69] Schneider CA, Rasband WS, Eliceiri KW. NIH Image to ImageJ: 25 years of image analysis. Nat Methods. 2012;9:671–5. 22930834 10.1038/nmeth.2089 PMC5554542

[70] Bankhead P, Loughrey MB, Fernández JA, Dombrowski Y, McArt DG, Dunne PD, et al. QuPath: open source software for digital pathology image analysis. Sci Rep. 2017;7:16878–16878. 29203879 10.1038/s41598-017-17204-5 PMC5715110

[71] Hao Y, Hao S, Andersen-Nissen E, Mauck WM, Zheng S, Butler A, et al. Integrated analysis of multimodal single-cell data. Cell. 2021;184:3573–87.e29. 34062119 10.1016/j.cell.2021.04.048 PMC8238499

[72] Wherry EJ, Ha SJ, Kaech SM, Haining WN, Sarkar S, Kalia V, et al. Molecular signature of CD8+ T cell exhaustion during chronic viral infection. Immunity. 2007;27:670–84. 17950003 10.1016/j.immuni.2007.09.006

[73] Wu T, Hu E, Xu S, Chen M, Guo P, Dai Z, et al. clusterProfiler 4.0: a universal enrichment tool for interpreting omics data. Innovation. 2021;2:100141. 34557778 10.1016/j.xinn.2021.100141 PMC8454663

[74] Trapnell C, Cacchiarelli D, Grimsby J, Pokharel P, Li S, Morse M, et al. The dynamics and regulators of cell fate decisions are revealed by pseudotemporal ordering of single cells. Nat Biotechnol. 2014;32:381–6. 24658644 10.1038/nbt.2859 PMC4122333

[75] Qiu X, Mao Q, Tang Y, Wang L, Chawla R, Pliner HA, et al. Reversed graph embedding resolves complex single-cell trajectories. Nat Methods. 2017;14:979–82. 28825705 10.1038/nmeth.4402 PMC5764547

[76] Cao J, Spielmann M, Qiu X, Huang X, Ibrahim DM, Hill AJ, et al. The single-cell transcriptional landscape of mammalian organogenesis. Nature. 2019;566:496–502. 30787437 10.1038/s41586-019-0969-x PMC6434952

