# Supplementary material for: Tissue-resident memory T cells break tolerance to renal autoantigens and orchestrate immune-mediated nephritis
Source: Cell Mol Immunol. 2024 Jul 3;21(9):1066–81. doi: 10.1038/s41423-024-01197-z (PMC11364874; doi:10.1038/s41423-024-01197-z)
Supplement: Supplementary file 1 — Supplementary Material [file 41423_2024_1197_MOESM1_ESM.pdf]

Supplementary Material for

Tissue-resident memory T cells break tolerance to renal autoantigens and orchestrate immune-mediated nephritis

Frederic Arnold, Laurence Kupferschmid, Philipp Weissenborn, Lukas Heldmann, Jonas F. Hummel, Paulina Zareba, Sagar, Manuel Rogg, Christoph Schell, Yakup Tanriver

Supplementary Material

Supplementary Figures S1 – S13

Supplementary Tables S1 – S5

References

Supplementary Figures

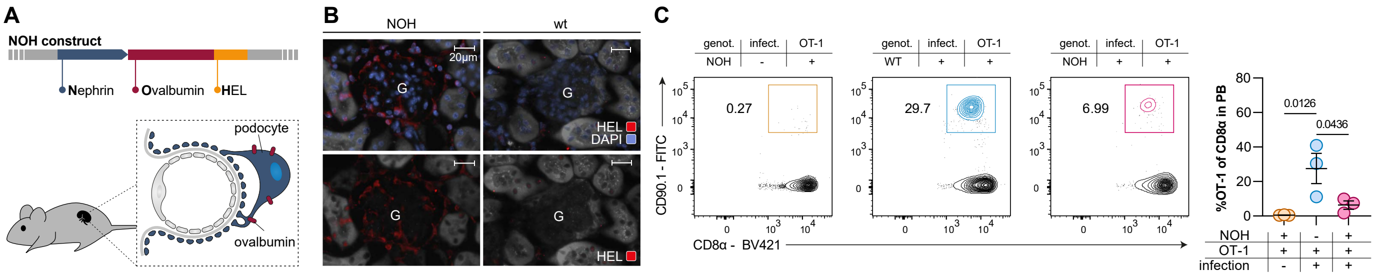

Figure S1. **(A)** Schematic showing the Nephrin-Ovalbumin-Hen egg lysozyme (NOH) construct. The nephrin promotor induces a podocyte-specific expression of the construct. The sketch below illustrates antigen-presentation. SIINFEKL ($OVA_{257\text{-}264}$) is presented on H-$2K^b$ restricted MHC class I histocompatibility complexes on podocytes. **(B)** Hen egg lysozyme (HEL; red channel) staining demonstrates expression of the NOH construct in podocytes. **(C)** Representative flow cytometry plots (left) and corresponding quantification (dot plot, right), identifying OT-1 cells ($CD8^+CD90.1^+$ gate) in peripheral blood. Yellow: NOH mice after OT-1 cell transfer, no infection. Blue: wt mice after adoptive OT-1 cell transfer and consecutive LM-OVA infection after 24 hours. Red: NOH mice after adoptive OT-1 cell transfer and consecutive LM-OVA infection after 24 hours. Analysis performed at day 7. Bars and whiskers of dot plots depict means and respective standard errors of the mean (SEM). P-values were calculated using one-way ANOVA with post hoc Tukey's multiple comparisons method.

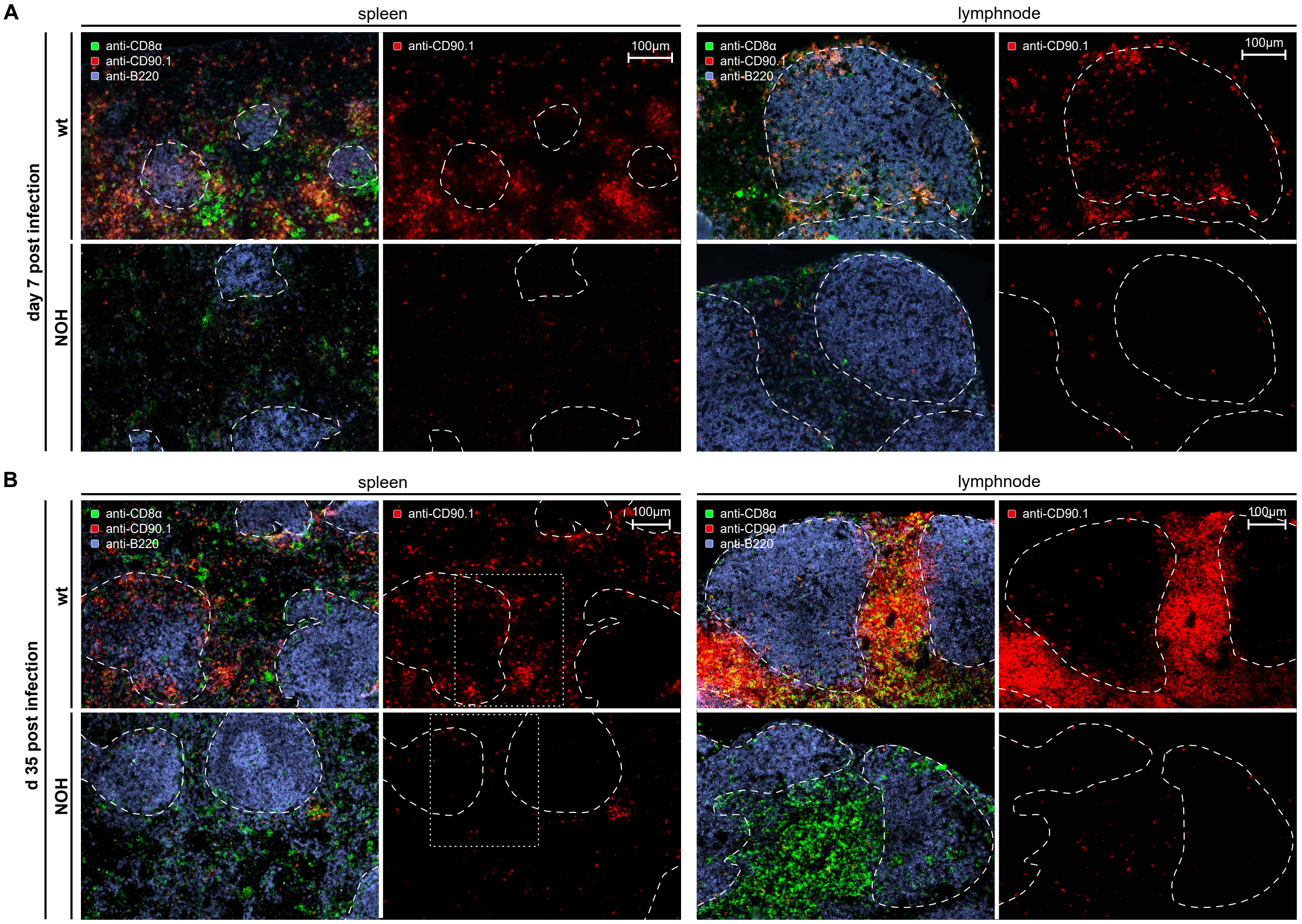

Figure S2. **(A, B)** Immunofluorescence of spleen (left panel) and lymph node sections (right panel), labelled with FITC-anti-CD8- (green channel), PE-anti-CD90.1- (red channel), and APC-B220- antibodies (blue channel), showing less of the adoptively transferred OT-1 CD8+ T cells in secondary lymphoid organs of NOH (lower row) *vs.* wt mice (upper row) after 7 (A) and 35 (B) days. Dashed lines mark B cell zones. Dotted boxes mark cutouts presented in Fig. 1.

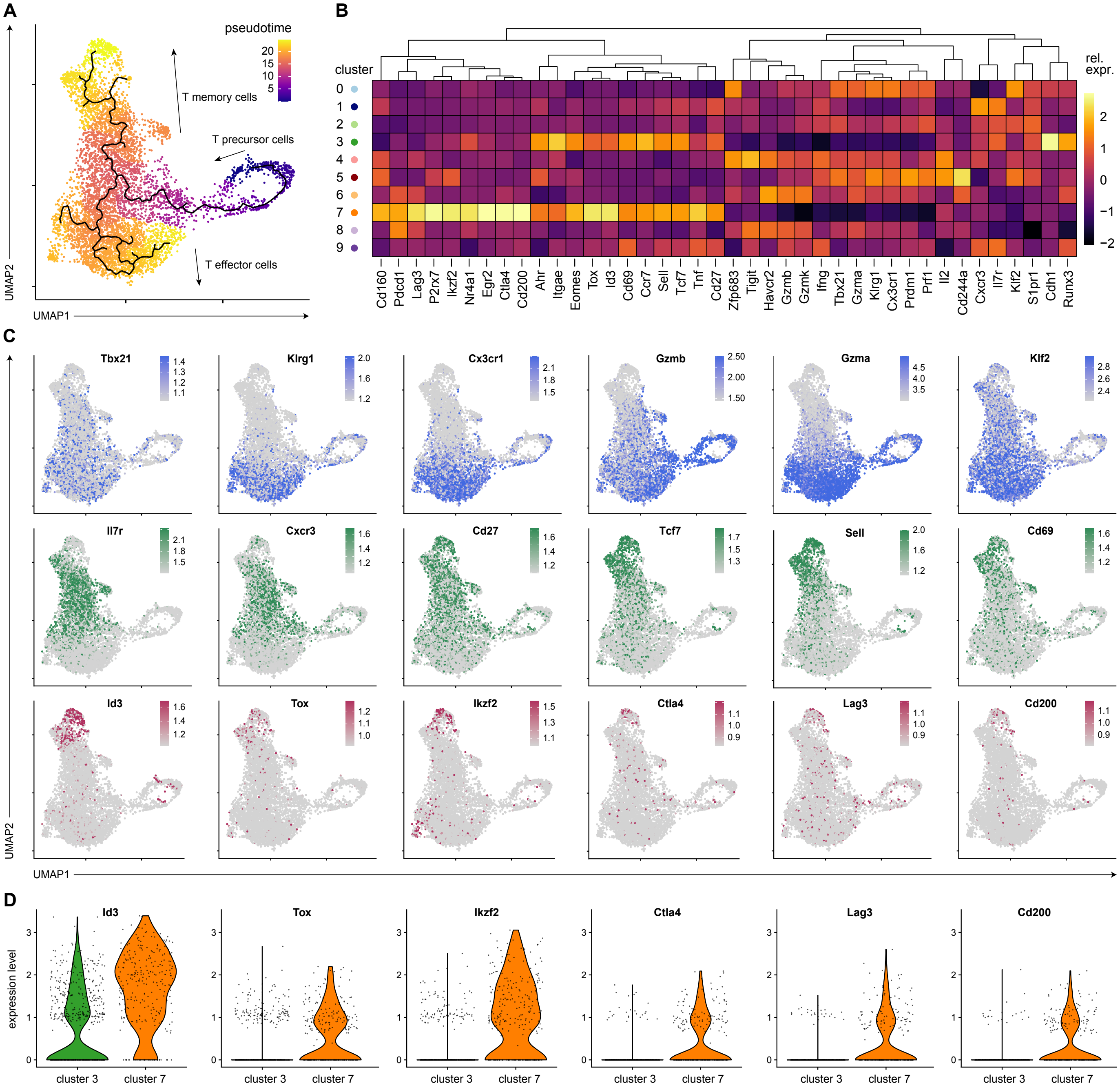

Figure S3. **(A)** Pseudotemporal ordering of single cells indicating differentiation of T effector (bottom) and T memory (top) cells from common precursors. **(B)** Heatmap illustrating relative expression of curated features. **(C)** UMAP feature plots showing expression of curated T cell activation/effector (upper row, blue), memory (central row, green) and inhibitory (lower row, red) markers (corresponding to Fig. 1, K and L). **(D)** Differential expression of selected T cell inhibitory genes comparing clusters 3 and 7. Corresponding to Fig. 1L.

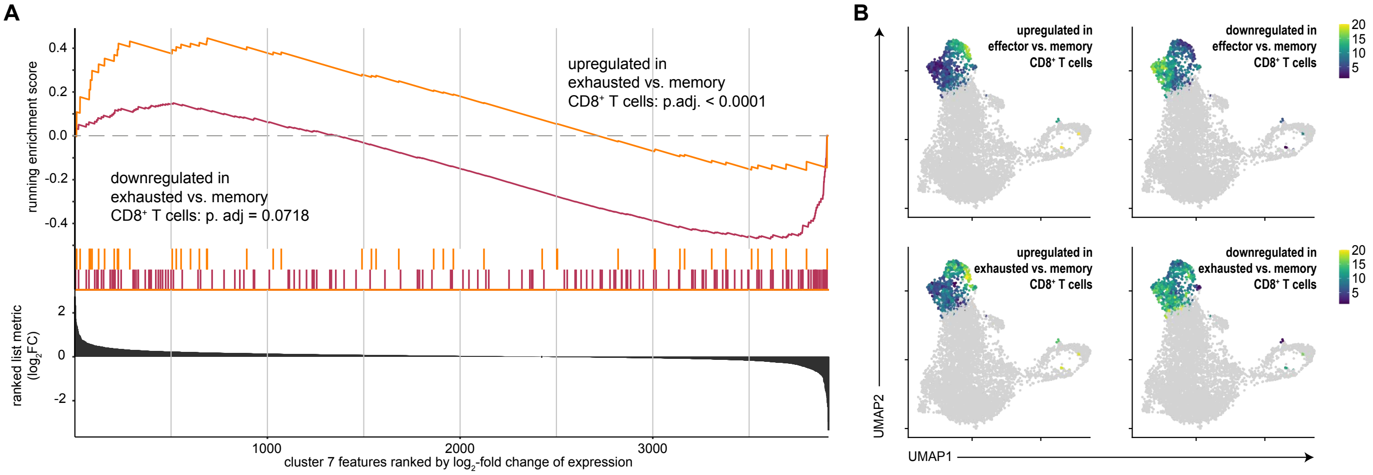

Figure S4. **(A)** Gene set enrichment plot for ranked expression of cluster 7 features, enriched in GSE9650 exhausted *vs.* memory CD8+ T cell analysis. Graphs show the running enrichment score (ES), barcodes the positions of gene set members on the rank-ordered gene list. **(B)** Feature expression plots depicting the UCell single-cell gene signature scoring of the GSE9650 effector *vs.* memory CD8+ T cell analysis (upper row) and the GSE9650 exhausted *vs.* memory CD8+ T cell analysis in clusters 3 and 7.

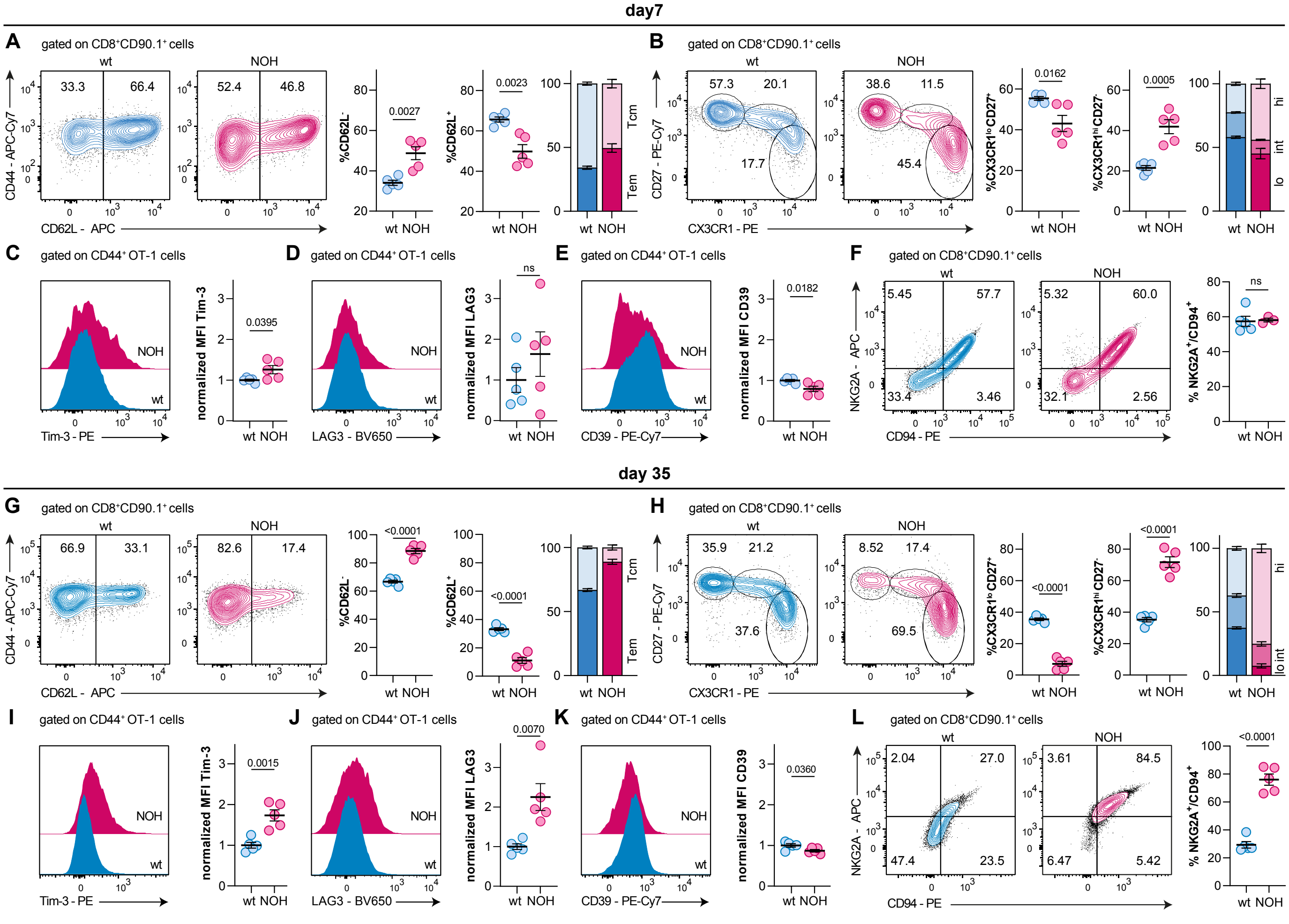

Figure S5. Flow cytometry analysis of splenocytes 7 **(A-F)** and 35 days **(G-L)** after adoptive OT-1 cell transfer and infection. **(A, G)** Representative flow cytometry plots illustrating CD44 and CD62L expression of transferred OT-1 cells. Dot plots and stacked bar plot depicting higher percentage of CD62L- T effector memory (T_{EM}) OT-1 cells in NOH (red) *vs.* wt mice (blue) on day 7 (A) and day 35 (G). **(B, H)** Representative flow cytometry plots illustrating CD27 and CX3CR1 expression of transferred OT-1 cells. Dot plots and stacked bar plot depicting higher percentage of CX3CR1hiCD27- OT-1 cells in NOH (red) *vs.* wt mice (blue) on day

7 (B) and day 35 (H). **(C, I)** Representative histograms illustrating Tim-3 expression of transferred OT-1 cells. Dot plot comparing normalized MFIs, showing higher Tim-3 expression in OT-1 cells from NOH (red) *vs.* wt mice (blue) on day 7 (C) and day 35 (I). **(D, J)** Representative histograms illustrating LAG3 expression of transferred OT-1 cells. Dot plot comparing normalized MFIs, showing higher LAG3 expression in OT-1 cells from NOH (red) *vs.* wt mice (blue) only on day 35 (J). **(E, K)** Representative histograms illustrating CD39 expression of transferred OT-1 cells. Dot plot comparing normalized MFIs, showing lower CD39 expression in OT-1 cells from NOH (red) *vs.* wt mice (blue) on day 7 (E) and day 35 (K). **(F, L)** Representative flow cytometry plots illustrating expression of CD94/NKG2A complex on transferred OT-1 cells. Dot plot showing higher expression in OT-1 cells from NOH (red) *vs.* wt mice (blue) only on day 35 (L). Bars and whiskers of dot plots depict means and respective standard errors of the mean (SEM). P-values were calculated using unpaired student's t test. All presented datasets are representative of at least 3 individual experiments with *n*=5 mice per group. Normalized MFIs were calculated by dividing the MFIs of each sample by the mean MFI of the respective wt group.

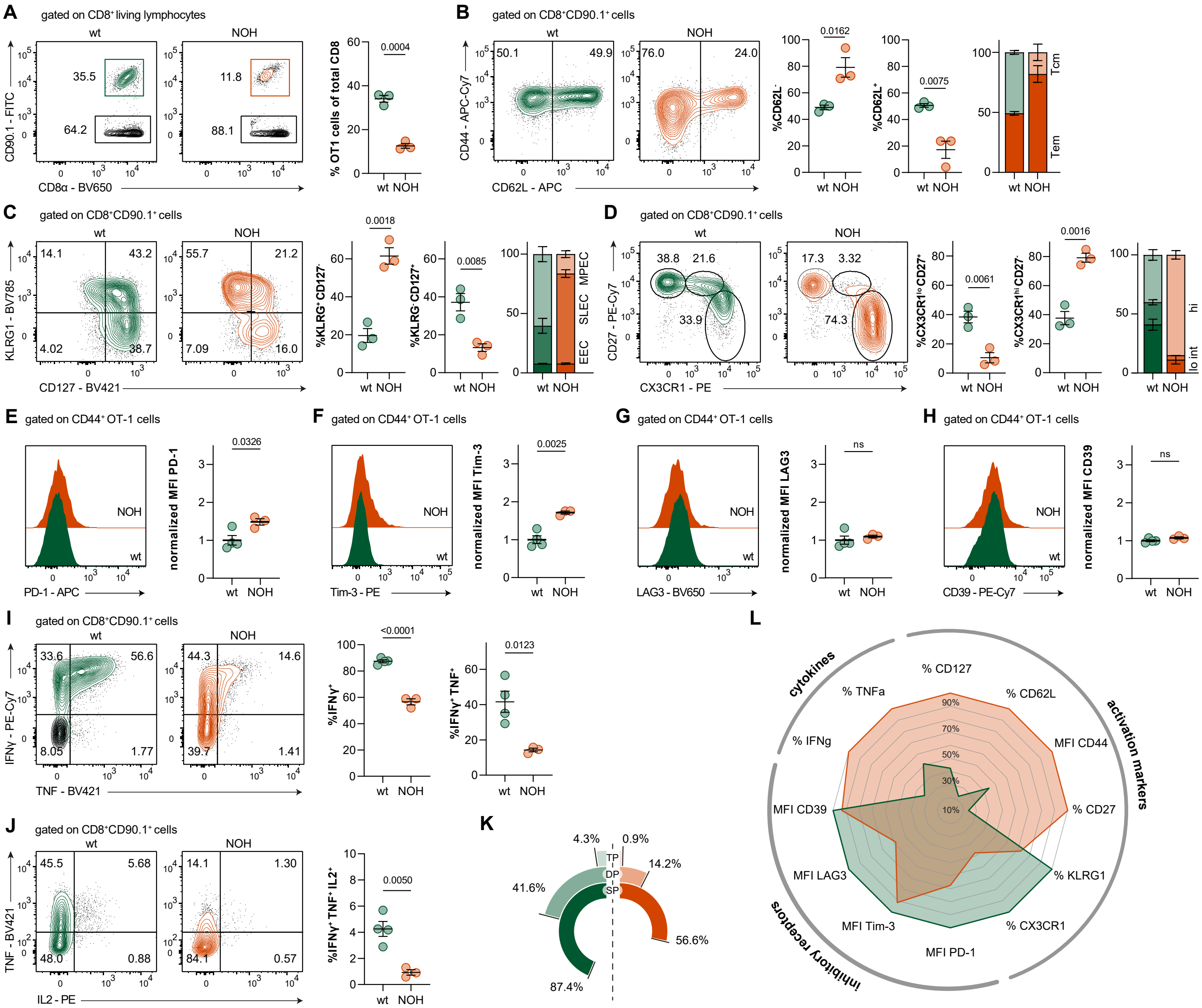

Figure S6. Flow cytometry analysis of splenocytes 35 days after OT-1 transfer and homologous prime-boost infection (d0: 2,500 cfu LM-OVA; d21: 250,000 cfu LM-OVA). **(A)** Representative flow cytometry plots discriminating transferred OT-1 cells from native CD8^{+} T cells (CD90.1^{-}) in splenocyte suspensions. Dot plot (right) showing significantly lower OT-1 cell fractions in NOH (orange) compared to wt mice (green), 35 days after homologous prime-boost infection. **(B)** Representative flow cytometry plots illustrating CD44 and CD62L expression of transferred OT-1 cells. Dot plots and stacked bar plot depicting higher percentage of CD62L^{-} T effector memory (T_{EM}) OT-1 cells in NOH (orange) *vs.* wt mice (green). **(C)** Representative flow cytometry plots illustrating KLRG1 and CD127 expression of transferred OT-1 cells. Dot plot and stacked bar plot depicting higher percentage of KLRG1^{+} CD127^{-} short lived effector OT-1 cells, compared to KLRG1^{-} CD127^{+} memory precursor OT-1 cells in NOH (orange) *vs.* wt mice (green). **(D)** Representative flow cytometry plots illustrating CD27 and CX3CR1 expression of transferred OT-1 cells. Dot plots and stacked bar plot depicting higher percentage of CX3CR1^{hi}CD27^{-} OT-1 cells in NOH (orange) *vs.* wt mice (green). **(E)** Representative

histograms illustrating PD-1 expression of transferred OT-1 cells. Dot plot comparing normalized MFIs, showing higher PD-1 expression in OT-1 cells from NOH (orange) *vs.* wt mice (green). **(F)** Representative histograms illustrating Tim-3 expression of transferred OT-1 cells. Dot plot comparing normalized MFIs, showing higher Tim-3 expression in OT-1 cells from NOH (orange) *vs.* wt mice (green). **(G)** Representative histograms illustrating LAG3 expression of transferred OT-1 cells. Dot plot comparing normalized MFIs, showing no significant difference of LAG3 expression in OT-1 cells from NOH (orange) *vs.* wt mice (green). **(H)** Representative histograms illustrating CD39 expression of transferred OT-1 cells. Dot plot comparing normalized MFIs, showing no significant difference of CD39 expression in OT-1 cells from NOH (orange) *vs.* wt mice (green). **(I)** Flow cytometry plots illustrating interferon-γ (IFNγ) and tumor necrosis factor (TNF) production of transferred OT-1 cells after *in vitro* restimulation with 10^{-6} M SIINFEKL peptide; cytokine excretion blocked with brefeldin A. Dot plots showing lower fractions of $IFN\gamma^{+}$ and $IFN\gamma^{+}TNF^{+}$ double positive OT-1 cells in NOH (orange) *vs.* wt mice (green). **(J)** Flow cytometry plots illustrating tumor necrosis factor (TNF) and IL2 production of transferred $IFN\gamma^{+}$ OT-1 cells after *in vitro* restimulation. Dot plots indicating lower fractions of $IL2^{+}IFN\gamma^{+}TNF^{+}$ triple positive OT-1 cells in NOH (orange) *vs.* wt mice (green). **(K)** Radial charts comparing cytokine production of OT-1 cells in NOH (orange) *vs.* wt mice (green). SP: single positive ($IFN\gamma^{+}$); DP: double positive, ($IFN\gamma^{+}TNF^{+}$); TP: triple positive: ($IL2^{+}IFN\gamma^{+}TNF^{+}$). **(L)** Spider graph summarizing relative expression of activation markers, inhibitory receptors and cytokine production. Bars and whiskers of dot plots depict means and respective standard errors of the mean (SEM). P-values were calculated using unpaired student's t test. All presented datasets are representative of at least 3 individual experiments with $n \geq 3$ mice per group. Normalized MFIs were calculated by dividing the MFIs of each sample by the mean MFI of the respective wt group.

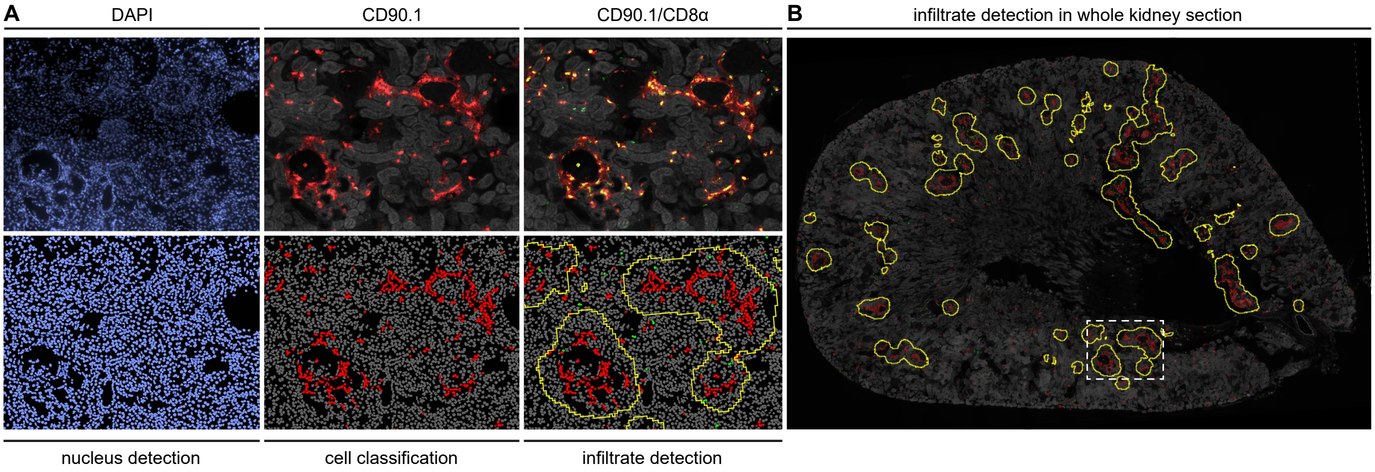

<u>Figure S7.</u> **(A)** Semi-automatic detection of renal T cell infiltrates. Panel on the left depicts identification of cells by nuclear DAPI staining (blue channel; left), classification of OT-1 cells by CD90.1 staining (red channel; center), and automatic detection of infiltrated areas (yellow outlines; right) with cluster detection algorithm implemented in QuPath. **(B)** Histopathology with marked infiltrated areas in an exemplary whole kidney slide of an NOH mouse 35 days after OT-1 transfer and heterologous infection (experimental setup as illustrated in Fig. 1A). Dashed white box marks cut-out shown in (A).

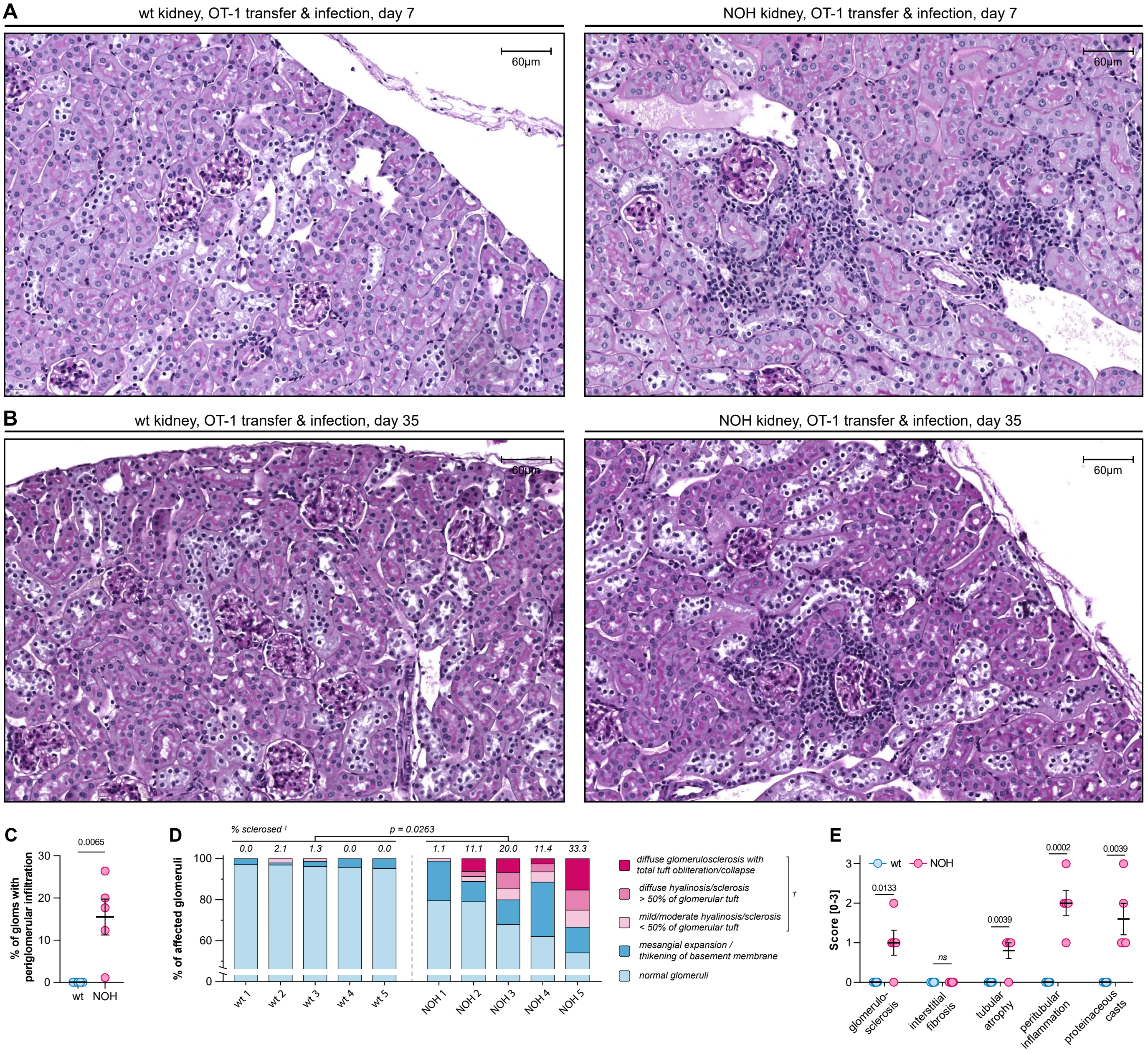

Figure S8. **(A, B)** Periodic acid–Schiff (PAS) stained kidney sections of wt (left) and NOH mice (right), 7 (A) and 35 days (B) after OT-1 cell transfer and infection, showing structural tissue properties (corresponding to Fig. 3). **(C)** Dot plot depicting percentage of glomeruli with surrounding infiltrations per whole kidney section. **(D)** Bar plot showing individual fractions of sclerosed glomeruli and severity of sclerosis after 35 days. Scoring was performed on whole kidney section as described in figure legend (*76*). **(E)** Combined dot plot comparing histopathological features of structural kidney damage of wt (blue) and NOH (red) mice after 35 days. The features glomerulosclerosis, interstitial fibrosis, tubular atrophy, and peritubular inflammation were scored by fractions of affected glomeruli or cortical area in whole kidney sections, respectively (0: <5%, 1: 5-25%, 2: 25-50%, 3: >50%). Proteinaceous casts were scored by their relative frequency (0: negative, 1: low, 2: moderate, 3: high) in whole kidney sections.

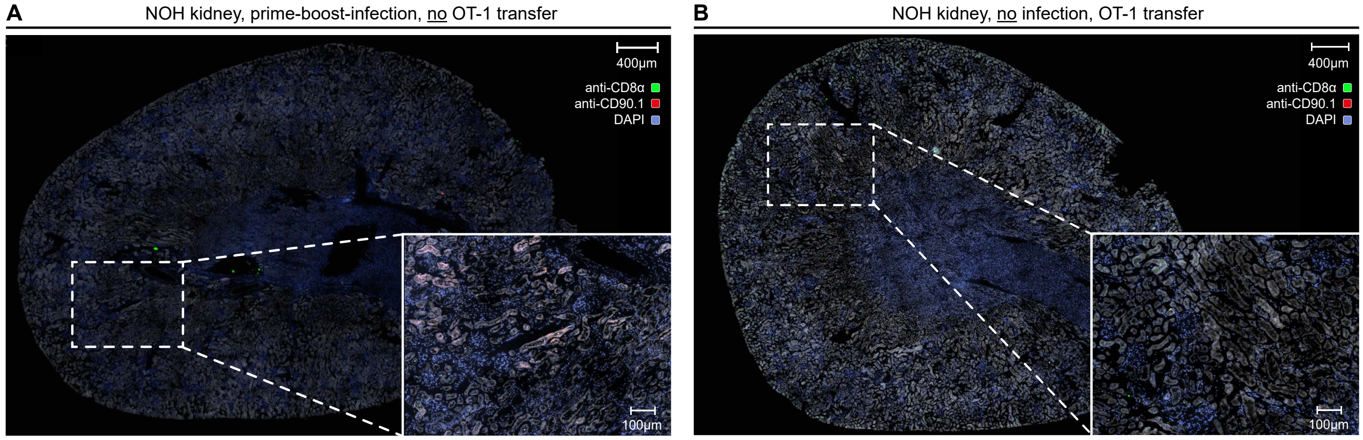

Figure S9. **(A)** Immunofluorescence of whole kidney sections, labelled with FITC-anti-CD8- (green channel) and PE-anti-CD90.1-antibodies (red channel), showing no T cells in NOH mice after prime-boost-infection without prior OT-1 transfer. **(B)** Immunofluorescence of whole kidney sections, labelled with FITC-anti-CD8- (green channel) and PE-anti-CD90.1-antibodies (red channel), showing no T cells in NOH mice after OT-1 cell transfer without infection.

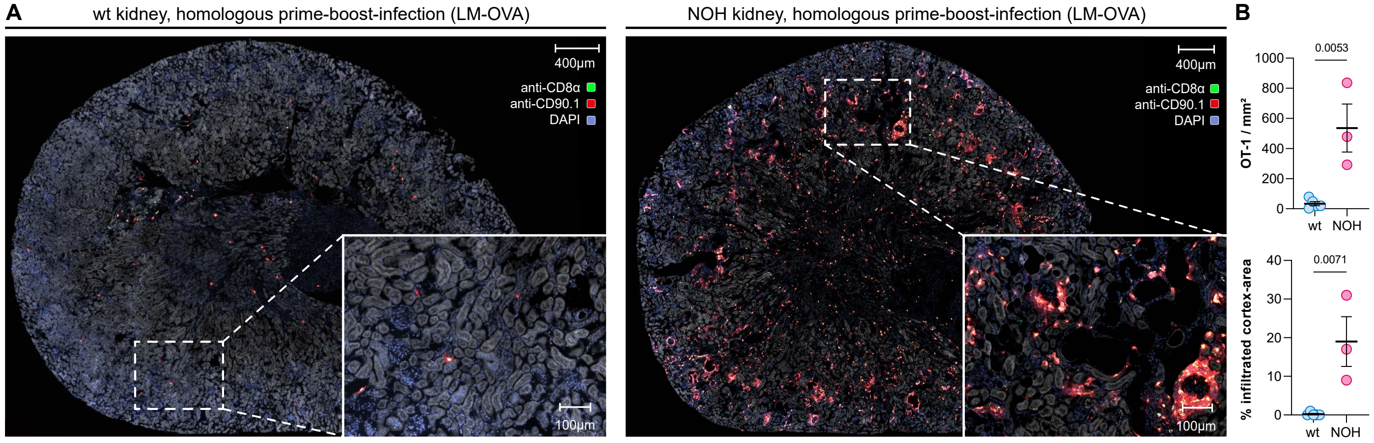

Figure S10. **(A)** Immunofluorescence of whole kidney sections, labelled with FITC-anti-CD8- (green channel) and PE-anti-CD90.1-antibodies (red channel), depicting the CD8$^+$ T cell infiltration in wt (left) and NOH mice (right) 35 days after OT-1 cell transfer, followed by an homologous prime-boost infection with LM-OVA (day 0: 2,500 cfu; day 21: 250,000 cfu). **(B)** The OT-1 cell density per mm^2 (upper graph) and the percentage of infiltrated cortex-are (lower-graph) are higher in NOH (red) *vs.* wt mice (blue) on day 35. Bars and whiskers of dot plots depict means and respective standard errors of the mean (SEM). P-values were calculated using unpaired student's t test. All presented datasets are representative of at least 3 individual experiments with $n \geq 3$ mice per group.

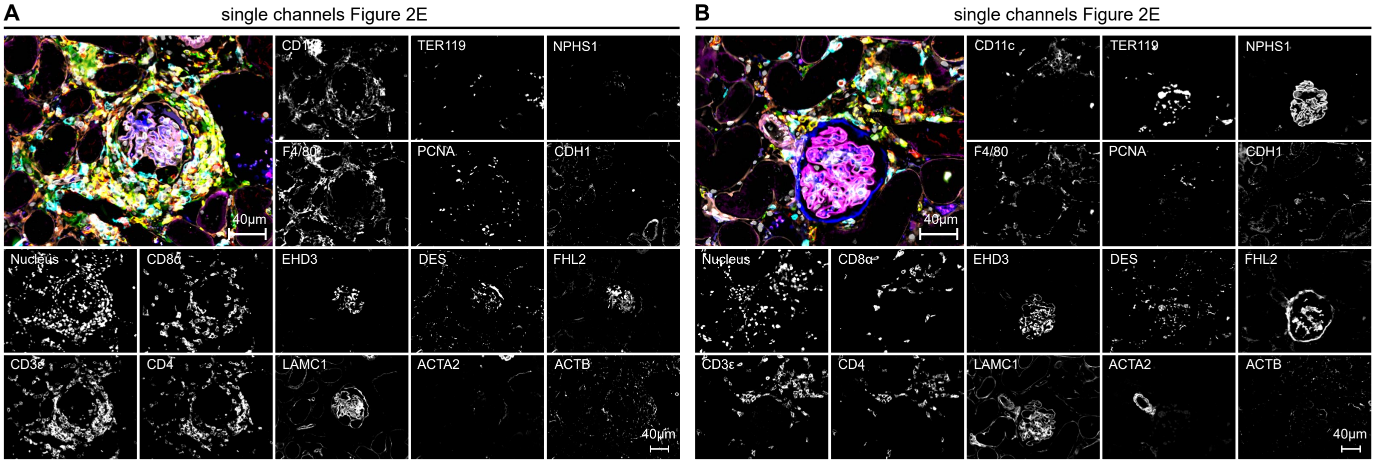

Figure S11. Single channels of 16-plex 4i immunofluorescence sections, stained for immune cell entities and structural markers. Corresponding to Fig. 3I.

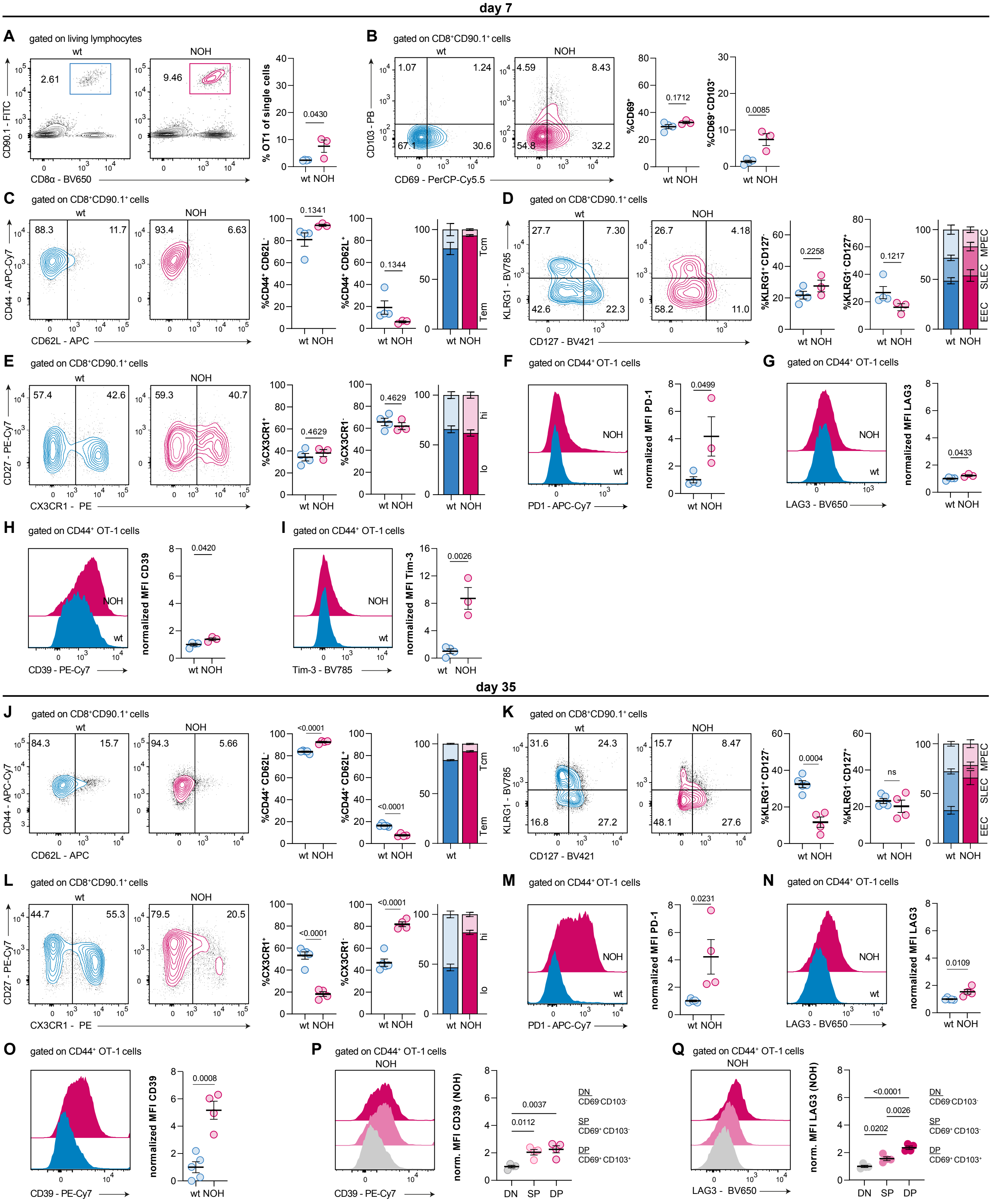

Figure S12. Flow cytometry analysis of kidney single cell suspensions 7 days **(A-I)** and 35 days **(J-Q)** after adoptive OT-1 cell transfer and infection. **(A)** Representative flow cytometry plots identifying renal OT-1 cells. Dot plot (right) showing higher percentage of OT-1 CD8⁺ T cells in kidneys of NOH (red) *vs.* wt mice (blue) on day 7. **(B)** Flow cytometry plots illustrating CD69 and CD103 expression of renal OT-1 cells. Dot plots depicting percentage of CD69⁺ cells (left) and double positive CD69⁺CD103⁺ OT-1 CD8⁺ T_{RM} cells (right) in kidneys of NOH (red) *vs.* wt mice (blue) on day 7. **(C, J)** Representative flow cytometry plots

illustrating CD44 and CD62L expression of renal OT-1 cells on day 7 (C) and day 35 (J). Dot plots and stacked bar plot depicting higher percentage of CD62L$^-$ T effector memory (T_{EM}) OT-1 cells in NOH (red) *vs.* wt mice (blue). **(D, K)** Representative flow cytometry plots illustrating KLRG1 and CD127 expression of renal OT-1 cells on day 7 (D) and day 35 (K). Dot plots and stacked bar plot depicting a lower percentage of KLRG1$^+$CD127$^-$ short lived effector OT-1 cells in NOH (red) *vs.* wt mice (blue) only on day 35. **(E, L)** Representative flow cytometry plots illustrating CD27 and CX3CR1 expression of renal OT-1 cells on day 7 (E) and 35 (L). Dot plots and stacked bar plot depicting a lower percentage of CX3CR1^{hi} OT-1 cells in NOH (red) *vs.* wt mice (blue) only on day 35. **(F, M)** Representative histograms illustrating PD-1 expression of renal OT-1 cells on day 7 (F) and 35 (M). Dot plot comparing normalized MFIs, showing higher PD-1 expression in OT-1 cells from NOH (red) *vs.* wt mice (blue). **(G, N)** Representative histograms illustrating LAG3 expression of renal OT-1 cells on day 7 (G) and 35 (N). Dot plot comparing normalized MFIs, showing higher LAG3 expression in OT-1 cells from NOH (red) *vs.* wt mice (blue). **(H, O)** Representative histograms illustrating CD39 expression of renal OT-1 cells on day 7 (H) and 35 (O). Dot plot comparing normalized MFIs, showing higher CD39 expression in OT-1 cells from NOH (red) *vs.* wt mice (blue). **(I)** Representative histograms illustrating Tim-3 expression of renal OT-1 cells on day 7. Dot plot comparing normalized MFIs, showing higher Tim-3 expression in OT-1 cells from NOH (red) *vs.* wt mice (blue). **(P)** Representative histograms illustrating CD39 expression of double positive (CD69$^+$CD103$^+$), single positive (CD69$^+$CD103$^-$) and double negative (CD69$^-$CD103$^-$) renal OT-1 cells in NOH mice on day 35. Dot plot comparing normalized mean fluorescent intensities (MFIs) of the CD39 signal, showing higher CD39 expression in double positive (CD69$^+$CD103$^+$) and single positive (CD69$^+$CD103$^-$) *vs.* double negative (CD69$^-$CD103$^-$) renal OT-1 cells. **(Q)** Representative histograms illustrating LAG3 expression of double positive (CD69$^+$CD103$^+$), single positive (CD69$^+$CD103$^-$) and double negative (CD69$^-$CD103$^-$) renal OT-1 cells in NOH mice on day 35. Dot plot comparing normalized mean fluorescent intensities (MFIs) of the LAG3 signal, showing higher LAG3 expression in double positive (CD69$^+$CD103$^+$) *vs.* single positive (CD69$^+$CD103$^-$) and double negative (CD69$^-$CD103$^-$) renal OT-1 cells. Bars and whiskers of dot plots depict means and respective standard errors of the mean (SEM). P-values were calculated using unpaired student's t test or one-way ANOVA with post hoc Tukey's multiple comparisons method if two or more groups were analyzed. All presented datasets are representative of at least 3 individual experiments with $n \geq 3$ mice per group. Normalized MFIs were calculated by dividing the MFIs of each sample by the mean MFI of the respective wt group.

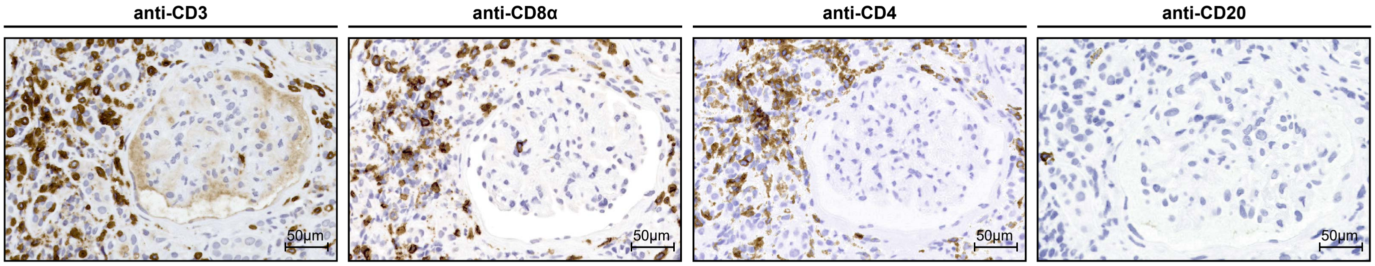

Figure S13. Periglomerular infiltrate in a human sample of immune checkpoint inhibitor (ICI) -associated nephritis. T cell infiltrate consists of CD8$^+$ CTLs and CD4$^+$ T helper cells. A relevant infiltration of B cells (CD20$^+$) was not detected.

Supplementary Tables

Fluorophore	Antibody (clone)	Dilution	Cat. number	Manufacturer
AF700	CD8β (YTS156.7.7)	1:1000	126618	BioLegend
APC	CD62L (MEL-14)	1:200	104412	BioLegend
	CD8α (53-6.7)	1:1000	120712	BioLegend
	MHC I H-2K^b bound to SIINFEKL (25D1.16)	1:100	141605	BioLegend
	CD159c/NKG2A (16A11)	1:200	142807	BioLegend
	TCRVβ5.1, 5.2 (MR9-4)	1:200	139505	BioLegend
	PD-1 (RMP1-30)	1:200	109117	BioLegend
APC-Cy7	CD44 (IM7)	1:500	103028	BioLegend
	PD-1 (29F.1A12)	1:100	135223	BioLegend
BV421 / PB	CD103 (2E7)	1:200	121421	BioLegend
	CD127 (A7R34)	1:100	135023	BioLegend
	CD8α (53-6.7)	1:500	100738	BioLegend
	CD90.1/Thy1.1 (OX-7)	1:500	202529	BioLegend
	TNF-α (MP6-XT22)	1:100	506328	BioLegend
BV510	CD44 (IM7)	1:800	103044	BioLegend
	CD8α (53-6.7)	1:400	100752	BioLegend
BV650	CD8α (53.6-7)	1:500	100741	BioLegend
	LAG3 (C9B7W)	1:50	125227	BioLegend
	MHC II I-A/I-E (M5/114.15.2)	1:1000	107641	BioLegend
BV785	CD4 (GK1.5)	1:500	100453	BioLegend
	KLRG1 (2F1)	1:100	138429	BioLegend
	Tim-3 (RMT3-23)	1:100	119725	BioLegend
FITC / AF488	CD326/Ep-Cam (G8.8)	1:1500	118210	BioLegend
	CD90.1/Thy1.1 (OX-7)	1:1000	202503	BioLegend
PE	CD44 (IM7)	1:500	103007	BioLegend
	CD69 (H1.2F3)	1:200	104507	BioLegend
	CD8α (53-6.7)	1:1000	100707	BioLegend
	CD90.1/Thy1.1 (OX-7)	1:1000	202524	BioLegend
	CD94 (18d3)	1:100	105507	BioLegend
	CD366 / TIM-3 (RM13-23)	1:100	119703	BioLegend
	CX3CR1 (SA011F11)	1:200	149006	BioLegend
	Il-2 (JES6-5H4)	1:100	503807	BioLegend
	TCR Vα2 (B20.1)	1:500	127808	BioLegend
PE-Cy7	CD11c (N418)	1:1000	25-0111-82	eBioscience
	CD27 (LG.3A10)	1:100	124216	BioLegend
	CD39 (Duha59)	1:100	143805	BioLegend
	CD45 (30-F11)	1:1000	25-0451-82	eBioscience
	IFN-γ (XMG1.2)	1:200	505826	BioLegend
PerCP-Cy5.5	CD69 (H1.2F3)	1:200	104521	BioLegend
Purified	Fc-block (αCD16/αCD32)	1:200		*self*

Table S1. Antibodies used for flow cytometry analysis.

Fluorophore	Antibody (clone)	Dilution	Cat. number	Manufacturer
APC / Cy5	B220 (RA3-6B2)	1:200	103226	BioLegend
	CD326/EpCam (G8.8)	1:200	118212	BioLegend
	CD8α (53-6.7)	1:200	120712	BioLegend
	CD90.1/Thy1.1 (OX-7)	1:200	202508	BioLegend
	MHC class II I-A/I-E (M5/114.15.2)	1:500	107613	BioLegend
FITC / AF488	B220 (RA3-6B2)	1:200	103228	BioLegend
	CD90.1/Thy1.1 (OX-7)	1:200	202503	BioLegend
	IgG (H+L) donkey anti-rabbit (polyclonal)	1:500	A21206	Life tech

Fluorophore	Antibody (clone)	Dilution	Cat. number	Manufacturer
PE / Cy3	CD90.1/Thy1.1 (OX-7)	1:500	202524	BioLegend
	CD103 (QA17A24)	1:200	156903	BioLegend
	CD274/PD-L1 (MIH6)	1:200	153611	BioLegend
	IgG (H+L) donkey anti-mouse (polyclonal)	1:500	715-165-151	Jackson IR
Purified	Anti-Lysozyme (polyclonal)	1:25	100-4172	Rockland

Table S2. Antibodies used for immunofluorescence imaging.

Antibody (clone)	Cat. number	Manufacturer
ACTA2 / SMA (1A4)	GA611	Agilent Dako
NPHS1 (polyclonal)	GP-N2	Progene
LAMC1 (EPR21199)	ab233389	Abcam
EHD3 (polyclonal)	HPA049986	Atlas Antibodies
E-Cadherin / CDH1 (4A2C7)	33-4000	Invitrogen
ACTB (AC-15)	A5441	Merck
PCNA (PC10)	M0879	Agilent Dako
FHL2 (polyclonal)	HPA006028	Atlas Antibodies
F4/80, (D2S9R)	70076	Cell Signalling
CD11c (D1V9Y)	97585	Cell Signalling
CD3ε (D4V8L)	99940	Cell Signalling
CD4 (D7D2Z)	25229	Cell Signalling
CD8α (D4W2Z)	98941	Cell Signalling
Desmin / DES (D33)	M0760	Agilent Dako
Ter199 (TER-119)	14-5921-82	Thermo Fisher

Table S3. Primary antibodies used for iterative indirect immunofluorescence imaging (4i).

Antibody (clone)	Cat. number	Manufacturer
CD3 (polyclonal)	IR503	Agilent Dako
CD4 (4B12)	IR649	Agilent Dako
CD8 (C8/144B)	IR623	Agilent Dako
CD20cy (L26)	IR604	Agilent Dako
CD69 (polyclonal)	HPA050525	Atlas Antibodies
CD103 (polyclonal)	HPA036313	Atlas Antibodies

Table S4. Antibodies used for immune histochemistry on human samples.

Antibody (clone)	Dilution	Cat. number	Manufacturer
B0301 α-mouse Hashtag 1 (M1/42; 30-F11)	0.5µg/10^6 cells	155831	BioLegend
B0302 α-mouse Hashtag 2 (M1/42; 30-F11)	0.5µg/10^6 cells	155833	BioLegend
B0303 α-mouse Hashtag 3 (M1/42; 30-F11)	0.5µg/10^6 cells	155835	BioLegend
B0304 α-mouse Hashtag 4 (M1/42; 30-F11)	0.5µg/10^6 cells	155837	BioLegend
B0305 α-mouse Hashtag 5 (M1/42; 30-F11)	0.5µg/10^6 cells	155839	BioLegend
B0306 α-mouse Hashtag 6 (M1/42; 30-F11)	0.5µg/10^6 cells	155831	BioLegend

Table S5. Hashtag antibodies used for single cell sequencing.

References

1. El Nahas A M, et al. Role of growth hormone in the development of experimental renal scarring. Kidney International. 1991;40: 29-34.
